# From statistical regularities in multisensory inputs to peripersonal space representation and body ownership: Insights from a neural network model

**DOI:** 10.1111/ejn.14981

**Published:** 2020-10-12

**Authors:** Tommaso Bertoni, Elisa Magosso, Andrea Serino

**Affiliations:** ^1^ MySpace Lab Department of Clinical Neuroscience Lausanne University Hospital (CHUV) University of Lausanne Lausanne Switzerland; ^2^ Department of Electrical, Electronic, and Information Engineering “Guglielmo Marconi” University of Bologna Cesena Italy

**Keywords:** bodily self‐consciousness, body representation, Hebbian learning, reference frame transformations, statistical inference

## Abstract

Peripersonal space (PPS), the interface between the self and the environment, is represented by a network of multisensory neurons with visual (or auditory) receptive fields anchored to specific body parts, and tactile receptive fields covering the same body parts. Neurophysiological and behavioural features of hand PPS representation have been previously modelled through a neural network constituted by one multisensory population integrating tactile inputs with visual/auditory external stimuli. Reference frame transformations were not explicitly modelled, as stimuli were encoded in pre‐computed hand‐centred coordinates. Here we present a novel model, aiming to overcome this limitation by including a proprioceptive population encoding hand position. We confirmed behaviourally the plausibility of the proposed architecture, showing that visuo‐proprioceptive information is integrated to enhance tactile processing on the hand. Moreover, the network's connectivity was spontaneously tuned through a Hebbian‐like mechanism, under two minimal assumptions. First, the plasticity rule was designed to learn the statistical regularities of visual, proprioceptive and tactile inputs. Second, such statistical regularities were simply those imposed by the body structure. The network learned to integrate proprioceptive and visual stimuli, and to compute their hand‐centred coordinates to predict tactile stimulation. Through the same mechanism, the network reproduced behavioural correlates of manipulations implicated in subjective body ownership: the invisible and the rubber hand illusion. We thus propose that PPS representation and body ownership may emerge through a unified neurocomputational process; the integration of multisensory information consistently with a model of the body in the environment, learned from the natural statistics of sensory inputs.

## Graphical abstract

Peripersonal space, the region of space immediately surrounding the body, is represented by multisensory neurons with visuo‐tactile responses in body‐part centred coordinates. The underlying reference frame transformations are crucial for body representation, and visuo‐tactile congruencies shape the sense of ownership for the body. We show how both can emerge in an artificial neural network model, based on Hebbian learning of the natural statistics of visual, proprioceptive and tactile inputs.

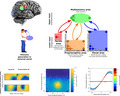

**Abbreviations**
HMD: head‐mounted display
IHI: Invisible Hand Illusion
PPS: peripersonal space
RBM: restricted Boltzmann machine
RF: receptive field
RHI: Rubber Hand Illusion
RT: reaction time
*SD*: standard deviation
VR: virtual reality

## 1 INTRODUCTION

### 1.1 Peripersonal space

Peripersonal space (PPS) is typically defined as the region of space immediately surrounding the body, or the space where we can physically interact with external objects, either actively, by reaching to touch them, or passively, when we enter in contact with an incoming object (di Pellegrino & Làdavas, 2015; Serino, 2019). PPS was originally defined in terms of a physical space, with a specific neural representation, following long‐known selective impairments of action and perception for stimuli in the near space induced by natural lesions in brain‐damaged patients (Brain, 1941) and by experimental lesions in monkeys (Rizzolatti et al., 1983). This concept was then expanded by neurophysiological and behavioural studies focusing on multisensory processing of stimuli within a limited distance from the body. In particular, studies on non‐human primates have described a population of multisensory neurons responding to visual and/or auditory stimuli, close to specific body parts, and to tactile stimulation of the same body parts (Duhamel et al., 1998; Graziano et al., 1994; Rizzolatti et al., 1981). That is, they present multisensory receptive fields which are selective for given body parts and anchored to them in space. Such evidence has been interpreted as the demonstration of the existence of a system representing the space around the different parts of the body in the primate brain, whose extent is defined by the extent of the multisensory receptive fields of those neurons. The term PPS then came to define not only a topographical region, but also its neural representation, leading to a variety of different descriptions whose common principles we try to resume here.

Evidence for the existence of an analogous system in humans comes from a body of neuropsychological (Farnè & Làdavas, 2002; di Pellegrino et al., 1997), behavioural (Spence et al., 2000; Zampini et al., 2007), and neuroimaging (Brozzoli et al., 2011; Grivaz et al., 2017; Makin et al., 2007) studies, coherently showing that interactions between tactile processing and visual and/or auditory cues is stronger when these stimuli are presented close to the body, as opposed to far.

Finally, several experimental results suggest to interpret PPS as a shell of interaction between the body and the environment, in which potential contacts between body parts and external objects are processed and predicted, with defensive (prepare reactions to potential threats) or appetitive (e.g., during reaching movements) purposes (Bufacchi & Iannetti, 2018; Cléry et al., 2015; Serino, 2019).

### 1.2 Previous models and motivation

Magosso, Ursino, et al. (2010) have developed a neural network model aiming to reproduce the main features of PPS representation in a neurophysiologically plausible computational framework. The model consists of two unisensory neuronal populations (auditory/visual, tactile), connected to a multisensory population: the receptive fields of visual/auditory neurons cover an extended space around the hand (or another target body part), while those in the tactile population code for touch on the same body part (Magosso, Ursino, et al., 2010; Magosso, Zavaglia, et al., 2010). In order to reproduce the space‐dependent responses of multisensory neurons in the PPS system, the connectivity of the network was tuned as follows: both tactile and visual/auditory neurons coding for stimuli that are close to the hand project strongly to the multisensory layer, whereas visual/auditory neurons coding for far stimuli project weakly to the multisensory layer. Thus, tactile stimuli on the body and visual/auditory stimuli close to the body induce stronger multisensory interaction than stimuli in the far space. This architecture reproduced neurophysiological (Bernasconi et al., 2018) and behavioural (Serino, Noel, et al., 2015) results of enhanced tactile processing in the presence of stimuli inside versus outside the PPS, and also of plastically induced changes in PPS representation (Magosso, Zavaglia, et al., 2010; Serino, Canzoneri, et al., 2015).

PPS representation is inherently body part centred. While tactile stimuli are directly processed in body‐centred reference frames, external auditory and visual stimuli are initially processed in head‐centred and eye‐centred reference frames. Thus, PPS representation requires a complex set of reference frame transformations on the incoming stimuli in order to estimate their position relative to the different body parts. For the sake of simplicity, the neural network model proposed by Magosso and colleagues assumed static body parts, as if reference frame transformations had been already achieved by means of other mechanisms. Other computational models have proposed to account for reference frame transformations, for instance by Pouget et al. (2002), and Makin et al. (2013). Pouget and colleagues modelled reference frame transformations by simulating three interconnected populations: two of them encode the position of the same stimulus in different reference frames, and the third encodes the offset between the two reference frames. For instance, one population could code for the visual (retinotopic) position of a stimulus, the second population for the auditory (head‐centred) position of the same stimulus, while the third could encode the shift between the two reference frames, represented by the gaze angle. By adjusting the weight of feedback and feedforward synapses, the model could either compute the position in a given reference frame based on the activity in the other two populations, or optimally integrate the three of them to increase the reliability of the information in each modality. However, it has not been investigated whether a similar model could also account for the emergence of body‐part centred visuo‐tactile interactions as the key property of PPS representation.

An additional limitation of the previous model is that the synaptic connections that underlie PPS representation in Magosso, Ursino, et al. (2010) work were hard‐wired, and while a second model (Magosso, Zavaglia, et al., 2010) adds Hebbian plasticity, this was only done on top of a pre‐defined synaptic connectivity. Therefore, existing models cannot explain how the spatial organization of the multisensory receptive fields underlying PPS representation emerges. Such neural representation has been shown to be highly plastic, e.g., it extends after using a tool to reach far portions of space (Canzoneri et al., 2013; Iriki et al., 1996; Maravita & Iriki, 2004). Interestingly, it was also shown behaviourally that PPS representation can be modified with simple audio‐far/tactile‐near stimulation, unrelated with tool use (Serino, Canzoneri, et al., 2015). It is therefore reasonable to suppose that that PPS representation might arise from networks of neurons whose large scale architecture, at the level of functional areas, is hard‐wired genetically in the brain, but in which the fine structure is based on the spontaneous tuning of synaptic connectivity induced by multisensory inputs through Hebbian learning. Hence, a key question in the field is not only to render how multisensory integration within overlapping visual and tactile receptive fields occurs, but also how such overlap is formed and maintained throughout development and everyday life.

### 1.3 Aim of the work

The aim of the present study is therefore to extend the previously established model of PPS representation (Magosso, Ursino, et al., 2010), in order to formalize a neurocomputational framework able to learn visuotactile associations from experience, and maintain them as body parts move in space. More specifically, with the model we aim to show:

1. How the synaptic connectivity that arises from natural stimulation in the environment can account for the emergence of overlapping visual/auditory and tactile receptive fields (RFs) subtending PPS representation.
2. That the same learned associations that build PPS representation implicitly perform reference frame transformations in body‐part centred coordinates. Therefore, a key novel point of our study is to demonstrate that PPS representation and reference frame transformations can emerge spontaneously and simultaneously within a unified neurocomputational process, by learning the statistical associations in multisensory inputs that occur naturally when interacting through the body within the environment. As a key example of PPS representation, here we focused on visuotactile integration around the hand, in hand‐centred reference frames.

To achieve our aims, we have adapted our previous model (Magosso, Ursino, et al., 2010) via two main modifications. First, proprioceptive inputs, previously neglected, were now taken into account by adding a population of proprioceptive neurons coding the location of the hand in space with respect to the trunk. Second, several psychophysical (Alais & Burr, 2004; Ernst & Banks, 2002), theoretical and computational works (Knill & Pouget, 2004; Ma et al., 2006; Makin et al., 2013) suggested to model multisensory integration in a probabilistic framework. This assumption guided us towards the choice of a plasticity rule designed to learn the statistical properties of visual, proprioceptive and tactile inputs. In the interest of approximating a key feature of biological neural networks that is key to our aims, we imposed the additional constraint that the learning rule should be Hebbian‐like, that is, based only on local correlations between neural activities. The network, still keeping the fundamental architecture of unisensory populations reciprocally connected with a multisensory layer, was therefore formalized as a Restricted Boltzmann Machine (RBM), a type of artificial neural network designed to efficiently learn the unknown joint probability distribution of its set of inputs through a local learning rule (Hinton & Salakhutdinov, 2006; Makin et al., 2013). Thus, we did not simulate the response to multisensory (tactile and visual) stimuli close to the hand through pre‐programmed synapses between the network's populations. Instead, we simulated a training where multisensory stimuli are randomly presented in space, with the only constraint, based on the physical properties of the body, that tactile inputs are simultaneously associated with visual inputs occurring on or near the hand, and never with far visual stimuli. We then let the model tune its synaptic connectivity to learn the statistical regularities in such a pattern of stimulation. This was compared with another “unconstrained” training model, where tactile and visual inputs were provided randomly and independently. We showed how, after the “body‐constrained” training, the model produces multisensory responses to tactile stimuli on the hand and visual stimuli close to the hand, as a function of the position of the hand in space, suggesting the emergence of multisensory, hand‐centred, receptive fields. Results from in silico computational simulations were then compared with results from in vivo psychophysical experiments to demonstrate the plausibility of the model. Finally, we also tested the model with analogue patterns of multisensory stimulation as those used to affect the sense of body ownership during the so‐called invisible hand illusion (IHI; Guterstam et al., 2013) and rubber hand illusion (Botvinick & Cohen, 1998). By measuring the network's response from the proprioceptive population, we could reproduce a computational analogue of the so‐called proprioceptive drift, i.e., a shift in the perceived location of one's own hand that is considered a behavioural proxy of changes in body ownership obtained via the illusions. Furthermore, we showed how the network's principles can be generalized to obtain similar results from more complex architectures. We included a visual population coding for hand position, changed the encoding schema of proprioceptive inputs to joint angles, and added another reference frame transformation, by encoding visual inputs in eye‐centred coordinates and adding a population coding for gaze angle.

## 2 MATERIALS AND METHODS

### 2.1 Qualitative network description

**FIGURE 1:**
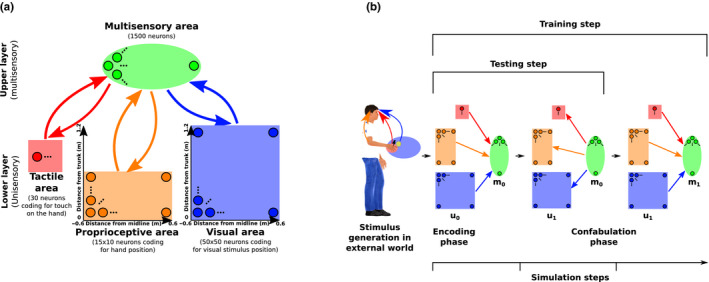
Network architecture, training and testing. (a) Architecture of the network. In the lower layer, three unisensory populations encode tactile stimulation on the hand, the proprioceptive position of the hand, the position of a visual stimulus. The upper layer is composed of multisensory neurons, in the sense that they receive inputs from each of the three unisensory populations. Each neuron in the proprioceptive and visual population has a preferred position distributed on a regular grid, with a Gaussian tuning curve of fixed width (~13 cm and ~11 cm respectively). For every stimulus, the number of spikes of neurons in the lower layer is drawn from a Poisson distribution, whose mean is determined by the tuning curve and a randomly selected gain in the range 4–10. The activity of neurons in the tactile population is set to 0 when the distance between the hand and the visual stimulus is greater than 15 cm. If the distance is smaller than 15 cm, the spike count for the tactile neurons is drawn from a Poisson distribution of mean 4–10, with this value randomly selected for each stimulus. Neurons in the lower layer are connected to neurons in the upper layer by bi‐directional, symmetric synapses. (b) One training/testing step of the network. During testing, one stimulus is generated and encoded in the lower layer (u_0_), and the activity of the upper layer (m_0_) is computed based on the unisensory neurons activity. Then, the activity of the unisensory neurons is re‐computed based on the multisensory neurons' activity to obtain the read‐out of the integrated information encoded in the multisensory population (u_1_). During training, an additional encoding step (confabulation phase) is added, where the activity of the multisensory neurons (m_1_) is computed based on the reconstructed activity in the unisensory populations (u_1_). Then, the synapses are updated with a weight change proportional to the difference in correlations between the lower and upper layer neurons in the two phases

While built upon Magosso, Ursino, et al. (2010) model architecture, the model presented here substantially differs from the previous one. First, in order to account for the evidence showing that the response of PPS neurons is modulated by proprioceptive inputs, in the present study we included a proprioceptive neural population coding for hand position, in addition to the two unisensory tactile and visual (or auditory) neural populations. Second, in order to overcome the necessity of hard‐wired synapses, and model the learning of reference frame transformations and PPS representation from synaptic tuning to external stimuli, we used a Restricted Boltzmann Machine (RBM) with two layers. RBMs are conceptually simple networks, widely used in unsupervised machine learning because of their efficiency in learning complex probability distributions. In their simplest form, they consist of two sets of units arranged in two layers, the lower layer and upper layer (usually called visible and hidden units respectively). A layer is defined as a pool of neurons that have no connections within the layer, but have bidirectional connections with neurons in the other layer. The units in the lower layer code for the components of an observation/event: in our RBM (Figure 1a), the lower layer is composed of the populations of unisensory neurons (proprioceptive, tactile, visual) that code the unisensory components of an event/stimulus. The units in the upper layer, (called multisensory layer in our RBM as it receives convergent inputs from multiple modalities) model the dependencies among these components. Even if there is no strict biological equivalent, the two layers can be seen as two levels in the processing of sensory information, where the lower layer receives the unisensory inputs, and the upper layer integrates them. We chose to restrict the network to two layers in our simpler model, for the sake of the interpretability of the results. Clearly, the sharp distinction between layers is a purely conceptual construct, and biological multisensory processing takes place in a more complex fashion, involving possibly more “layers” of recurrent processing. Nevertheless, our choice goes in the direction of showing that a simple architecture is general enough to capture the key features of multisensory integration in PPS. In the lower layer, unisensory tactile neurons code for touch on the hand, proprioceptive neurons code for the position of the hand with respect to the trunk, and visual neurons code for the position of an external stimulus in trunk‐centred coordinates (Figure 1a). Note that, for the visual population, this implies that inputs are represented as if the head and fixation were kept fixed, omitting for simplicity two additional components of the full reference frame transformation from retinotopic to head‐centred to body‐centred coordinates. Visual inputs are originally coded in eye‐centred reference frames. Thus, to gather proper information about the position of the visual stimuli with respect to the hand's position, visual inputs need to be recoded in more global, trunk centred reference frame. These transformations can be added to our model by including populations coding for head and eye positions, and letting the network learn the joint distribution over all the neural populations. In the last paragraph of the results section, we demonstrate how the main results of this work can be recovered from a network including a fourth population coding for gaze angle, therefore implementing an additional reference frame transformation. The upper layer consists of multisensory neurons, i.e. neurons that receive inputs from the three unisensory populations. The visual and proprioceptive populations represent areas of 1.2 × 1.2 and 1.2 × 0.6 meters in front of the trunk, respectively, with the first dimension representing the medial‐lateral axis and the second dimension the anterior‐posterior axis. The specified sizes refer to the area where stimuli are actually delivered during training, while the area spanned by the neurons' preferred positions is slightly larger due to the margins to prevent edge effects. Similarly to what was done in previous models of multisensory integration, visual and proprioceptive populations use a population coding with Gaussian tuning curves to encode the positions of the visual stimulus and of the hand (Ma et al., 2006). The tactile area simply encodes the presence of tactile stimulation by activating all its neurons with a mean value proportional to the stimulation intensity.

In order to gauge the unisensory inputs' parameters, we required the maximal theoretical visual and proprioceptive precision under an optimal decoder to be consistent with behavioural human studies (Jones et al., 2010; Rincon‐Gonzalez et al., 2011; Van Beers et al., 1998). Such value is defined as the standard deviation of the posterior probability of the stimulus location, given the activity of the unisensory population. It depends on the gain (i.e., the strength) of the stimuli and on the density of neurons per unit of space represented, and can be calculated with good approximation on the same bases as in previous works (Ma et al., 2006; Makin et al., 2013) (see Supporting Information for the detailed calculation). With the chosen parameters, the proprioceptive precision at maximal gain is 1.68 cm, and the visual accuracy is 0.45 cm, consistently with what has been reported in human behavioural studies (Jones et al., 2010; Rincon‐Gonzalez et al., 2011; Van Beers et al., 1998). The number of multisensory neurons was determined empirically, looking for the optimal trade‐off between minimizing the number of units and maximizing network performance. Specifically, the number of hidden units was set so that a further increase in their number would not lead to significant improvement in the precision of positions encoded in the multisensory layer (see Supporting Information for details). While the receptive fields of the unisensory populations are defined a priori, the receptive fields of the multisensory neurons are learned during training. As widely done in RBMs, we used one‐step contrastive divergence as learning algorithm. Contrastive divergence is based on local correlations between neuronal activity and does not require backpropagation, and can be therefore mapped to biologically realistic plasticity mechanisms, namely Hebbian learning. In machine‐learning, RBMs are used to learn a generative model of the probability distribution of the inputs presented during the training. This means that, after a successful training, samples taken from the spontaneous activity of the network should come from the same probability distribution as the training examples. Through this mechanism, RBMs have been used to model multisensory integration and reference frame transformations (Makin et al., 2013). Here we test the hypothesis that, in a similar way, the emergence of PPS representation can be simply modelled by letting a neural network learn the regularities of its sensory inputs, represented by correlations across different sensory modalities.

### 2.2 Mathematical network description

In a probabilistic population code, such as the one used for the generation of stimuli in our network, the activity of neurons in the unisensory populations can be seen as a probability distribution conditioned on the position of the stimuli in the physical world, from which spike counts are drawn for each population. Let **x_v_** be the (2D) position of the visual stimulus, and **x_p_** the position of the hand in the same 2D plane, then the activity of the *i*‐th unisensory neuron *u_i_* is defined by:

(1) $$u_{v_{i}}=Pois\left(\lambda_{v_{i}}\right),\lambda_{v_{i}}=g_{v}e^{‐\frac{{∥x_{v}‐{\hat{x}}_{v_{i}}∥}^{2}}{2\sigma_{v}^{2}}}$$

(2) $$u_{p_{i}}=Pois\left(\lambda_{p_{i}}\right),\lambda_{p_{i}}=g_{p}e^{‐\frac{{∥x_{v}‐{\hat{x}}_{v_{i}}∥}^{2}}{2\sigma_{p}^{2}}}$$

(3) $$u_{t_{i}}=Pois\left(g_{t}\right)\;\text{if}\;∥x_{v}‐x_{p}∥<0.15m,0\;\text{otherwise}$$

where *u_v_*, *u_p_*, *u_t_*, respectively, denote neurons belonging to visual, proprioceptive and tactile populations, and $\hat{x}$ denotes the preferred position of a given neuron. The *SD* of the tuning curves (*σ_v_* and *σ_p_*) was set at three neurons for the visual population, and at one neuron for the proprioceptive population (i.e., around 1/15 of the whole population's range, which gives ~13 cm for proprioceptive and ~11 cm for visual neurons). 30 tactile units are used, and the preferred positions of the proprioceptive and visual neurons tile the space on a regular grid of 15 × 10 and 50 × 50 neurons, respectively. This includes the 1.2 × 0.6 and 1.2 × 1.2 meters of space represented by the neural populations, plus a safety margin (approximatively three times the *SD* of the tuning curve, or 30 cm on each side in physical units) to avoid boundary effects. The width of the tuning curve was mainly determined during preliminary testing, on the basis of a set of heuristic criteria. We noticed that in order to allow efficient learning, the average learning signal from units from different populations needs to be approximately the same, hence the width of the tuning curve needs to be a fixed fraction of the total population range. Since the average firing rate of tactile units is fixed by the proportion of training inputs where touch is provided to approximately 5%, the width of visual and proprioceptive tuning curves was chosen to approximately match this value, while not requiring excessively large safety margins. In any case, the network's main predictions were robust with respect to the choice of such parameter, as shown in the Supporting Information. The parameter g represents the stimulus strength (gain), and is varied during training independently for each unisensory population, by drawing a random, uniformly distributed number between 4 and 10 for each stimulus presentation. Note that, alternatively, tactile inputs could have been encoded similarly to visual and proprioceptive inputs, with a population representing the whole hand whose individual neurons respond preferentially to specific locations. In preliminary testing, the two encoding schemas yielded largely overlapping results. However, the current encoding schema was preferred as empirical evidence shows that tactile receptive fields of PPS neurons tend to be large, covering whole body parts, suggesting that their functional role is to roughly predict tactile interaction at the level of entire body parts, more than predicting the specific location of tactile stimulation. Also note that the tactile population needs not be an early tactile area as S1, but possibly a higher level somatosensory area, responding prevalently to tactile stimulation.

The activity of neurons is updated simultaneously in all neurons in a given layer, based on the activity of neurons in the other layer. In other words, the network has no temporal dynamics, and, differently from the previous model, there are no intra‐layer connections, as the generation of spread‐out population level activation is simulated by the size of unisensory receptive fields. For simplicity, we define as an “up” pass when the activity of the upper layer is computed given the activity of the lower layer, and a “down” pass when the activity of the lower layer is computed given the activity of the upper layer. The up and down passes are defined as follows:

(4) $$Up:m=Bern\left(\mu \right),\mu =\sigma \left(\mathrm{Wu}+b_{m}\right),\sigma \left(x\right)=1/\left(1+e^{‐x}\right)$$

(5) $$Down:u=Pois\left(\lambda \right)\lambda =e^{W^{T}m+b_{v}}$$

where **u** is the vector of activity of all neurons in the lower layer (unisensory), **m** is the vector of activity of all neurons in the upper layer (multisensory), *W_ij_* is the synaptic weight connecting neuron m_i_ to neuron *u_j_*, and **b_u_** and **b_m_** are biases for unisensory and multisensory neurons respectively. Note that the fact that the matrix used in the “down” pass is the transpose of the matrix used in the “up” pass implies that feedforward and feedback synapses are symmetric. In practice, the multisensory neurons' activity, given the unisensory neurons' activity, is a vector of samples of Bernoulli variables, whose mean is a sigmoidal function of the weight matrix acting on the unisensory neurons. Conversely, the unisensory neurons' activity, given the multisensory neurons' activity, is a vector of samples of Poisson variables, whose mean is the exponential function of the weight matrix acting on the multisensory neurons. In RBMs, the choice of sigmoidal and exponential “link” functions is the standard for Bernoulli and Poisson units, respectively (Welling et al., 2004).

### 2.3 Training

The network was initialized with random connectivity, with each synaptic weight being drawn from a Gaussian with zero mean and 0.001 *SD*, and all biases were set to zero. Then, it was trained by presenting patterns of stimulations reproducing the natural associations between tactile, proprioceptive, and visual inputs. That is, for each training example, two independent, uniformly distributed positions were randomly generated for the hand and the visual stimulus, and encoded in the visual and proprioceptive populations, respectively. In the “body‐constrained” training, tactile stimulation was provided when the distance between the stimulus position and the hand position was smaller than 15 cm, roughly the centre to centre distance at which hand‐object tactile interactions are expected to take place. This resulted in tactile stimulation being provided in approximately 5% of the trials. In the control, unconstrained training, we randomly provided tactile stimulation in 5% of the trials, in order to remove the statistical regularity imposed by the body structure while keeping the amount of tactile stimulation constant. The input was encoded in the unisensory populations and then integrated in the upper layer through feedforward synapses, according to the rules defined in the previous paragraph. After this, a “confabulation” phase followed to complete the learning process for a given training example (Figure 1b). In the confabulation phase, the integrated stimulus was projected back to the lower layer through feedback connections, and again to the upper layer (Hinton, 2000). After a batch of 100 encoding‐confabulation sequences, the synaptic weight changes proportionally to the difference in the two phases in correlations between the upper and lower layer:

(6) $$\Delta W=\eta {〈u_{0}m_{0}‐u_{1}m_{1}〉}_{\mathrm{batch}}$$

(7) $$\Delta b_{u}=\eta {〈u_{0}‐u_{1}〉}_{\mathrm{batch}}$$

(8) $$\Delta b_{m}=\eta {〈m_{0}‐m_{1}〉}_{\mathrm{batch}}$$

where the subscript 0 indicates the activity after the first step of encoding the stimulus in the unisensory and multisensory layer, and the subscript 1 indicates the activity in the confabulation phase. It can be shown (Hinton, 2000) that this learning algorithm is approximately minimizing the information loss between the training data's probability distribution, and the lower layer's equilibrium probability distribution (that is, the distribution obtained after a sufficiently large number of up‐down iterations). In more neuroscientific terms, when the training is complete, the network's spontaneous activity should closely resemble the activity induced by sensory stimulation. Since this learning rule contains one positive and one negative term proportional to local correlations, this is an Hebbian‐anti‐Hebbian learning rule. The learning rate η was set to 0.005, and the training was run for 100 epochs in total, with each epoch consisting of 400 batches of 100 samples. The whole process took about two hours on a standard desktop computer.

### 2.4 Testing and simulating behaviour

After the training was completed, the network's features were assessed and compared to existing literature. While the receptive fields of the unisensory neurons in the lower layer are set a priori on the basis of prior knowledge from neurophysiological and computational studies, the receptive fields of multisensory neurons are learned during training, and can therefore be tested and compared with data from the literature. Moreover, the network was used to simulate behavioural experiments on multisensory integration, and the results were compared with behavioural data. In order to do so, it is necessary to establish a link between simulated neural activity and visuotactile interactions in behavioural experiments. The general procedure followed in this work was to decode the information contained in the multisensory layer after unisensory inputs are encoded together (i.e. integrated) in its shared representation. Since it would be very difficult to decode such information directly from the multisensory layer, we proceeded as Makin et al. (2013). In order to interpret the activity of multisensory units, their activity was projected down to the unisensory populations via a “down” pass through the feedback synapses (Figure 1b). Here, neural activity could be easily decoded, since the mapping between unisensory activity and the physical stimuli is defined a priori by the Gaussian tuning curves that we chose. It is sufficient to take the barycentre of the neural activity contained in the visual layer to decode the physical location of the visual stimulus encoded in the multisensory layer, the barycentre of the activity in the proprioceptive layer to decode the position of the hand with respect to the trunk, and, finally, the strength of the signal in the tactile layer to decode the intensity of tactile stimulation.

### 2.5 Behavioural experiments

#### 2.5.1 Rationale

The network uses a simplified set of sensory inputs, as visual information about the hand's position and appearance is not present. In literature, hand PPS representation in humans was typically assessed through a simple tactile detection task, in which in which reaction times to tactile stimuli on the hand are measured in the presence of task‐irrelevant auditory or visual stimuli, at various distances from the hand (Canzoneri et al., 2012). Using this paradigm, it was found that reaction times speed up (and tactile accuracy increases, as in Salomon et al., 2017) when the tactile stimulation is administered while the auditory or visual stimuli are closer to the body, with a stronger modulation in the case of looming stimuli. To our knowledge, visual information about hand position was always present in such experiments, and therefore the contribution of proprioception alone (simulated by the set of inputs of our model) was never assessed behaviourally. We therefore designed ad‐hoc experiments to test whether proprioceptive information alone can generate a hand‐centred PPS representation, that can be behaviourally detected through a tactile detection task. This was done by adapting the behavioural task described above to VR, allowing to keep the hand invisible while presenting visual stimuli close or far from its position in space.

#### 2.5.2 Materials

Tactile stimulation was delivered through rotating mass vibrators (Precision Microdrives), driven by a dedicated microcontroller. A hand‐held button was attached to the same microcontroller, in order to collect reaction times to tactile stimulation on the same device and minimize unpredictable delays. Visual stimuli were delivered in a virtual reality scenario. A Head Mounted Display (HMD, Oculus Rift) was used, and rendering of the virtual environment was performed through a custom made software (ExpyVR; http://lnco.epfl.ch/expyvr) coupled with the Steam VR software (SteamVR; https://www.steamvr.com/en).

#### 2.5.3 Participants

Forty‐three healthy participants (19 females, aged 25 ± 3.7 *SD*, ranging from 23 to 41 years) were recruited for the study, and received monetary compensation for their time. Only right‐handed participants with normal or corrected to normal vision were recruited for the study. The study conforms with the World Medical Association Declaration of Helsinki, was approved by the ethical committee of the Vaud canton, Switzerland, and was performed with the understanding and written consent of each subject.

#### 2.5.4 Procedure

Participants wore the HMD, and had two vibrators taped on the back of their right hand. They saw a virtual scenario reproducing a desk of the same size and location as the physical desk located in front of them, with a fixation cross located 15 cm above the desk and 65 cm in front of their trunk. They were instructed to keep their gaze on the fixation cross, and react as fast as possible when receiving tactile stimulation on the right hand, by pressing a button with the other hand, while trying not to pay attention to visual stimuli moving in their visual field.

#### 2.5.5 Design

The experiment used a within‐subjects design, with two hand positions, run in counterbalanced between‐subjects blocks. In “Hand right” blocks, participants placed their right palm on the desk about 30 cm in front of their trunk, and 25 cm right of their midline. In “Hand left” blocks, they placed the hand at the same distance from their trunk and 25 cm left of their midline. Within each block, four types of trials were present: three visuotactile trials and one unisensory. In visuotactile trials, participants saw a tennis ball starting from the fixation cross and moving at constant speed along one of three possible trajectories, directed towards one of three possible targets: “left,” corresponding to the hand position in the “Hand left” blocks, “right,” corresponding to the hand position in the “Hand right” blocks, and “receding,” corresponding to a point located on the midline around 30 cm in front of the fixation cross (see Figure 4a). Participants received a well above threshold 100 ms vibrotactile stimulus (both vibrators were activated at the same time) at one out of three randomized delays from trial onset, to reduce the predictability of tactile stimulation (1.75, 2 or 2.25 s from trial onset). The ball motion started 500 ms after trial onset, and lasted for 2 s at around 22.5 cm/s, so that tactile stimulation was received when the ball was either at 0, 5 or 10 cm from the target. In unisensory trials, the same scenario was displayed, and the tactile stimulus was administered with the same randomized delay, but no tennis ball was displayed. For each hand position block, a total of 21 trials per trajectory (of which 7 per delay) was collected for visuotactile trials, plus 21 unisensory trials. In addition, a total of 36 trials, 30% of the total, were catch trials. In such trials, one of the three usual ball trajectories was displayed (12 trials for each trajectory), but no tactile stimulation was delivered. Each experimental block lasted around 8 min.

#### 2.5.6 Data preprocessing and analysis

Reaction times (RTs) longer than 700 ms were automatically discarded by the microcontroller. This threshold can be considered safe as the average RT was 264.75 ms, with an average within‐subject *SD* of 33.9 ms, making it extremely unlikely to observe a true reaction time longer than 700 ms. Overall, subjects performed the task accurately, with 0.66% of omitted responses to tactile stimulation and 5.6% of false alarms (responses given in catch trials or before the stimulation). Such responses were discarded. We then removed outlier responses by discarding, for each subject and experimental block, RTs falling more than 2 median absolute deviations away from the median RT. This cut‐off is a more robust equivalent of the standard cut‐off at 2 SDs, as suggested by theoretical and empirical justifications in methodological work (Leys et al., 2013). The three randomized delays between 1.75 and 2.25 s were used purely to reduce the predictability of the task, and are orthogonal to the experimental conditions of interest. Seven trials for each delay were collected for each trajectory and block and the distance covered by the ball in the 500 ms randomization window is small compared to the distance between the three targets. This allowed us to overcome the possible confound introduced when the overt expectations due to the temporal delay of the tactile stimulation correlate with the position of the visual stimulus. Therefore, in our main analyses, we pooled trials from the three delays together, and only focused on the effects of hand position and ball trajectory. Similarly to what was done in previous studies (Serino, Canzoneri, et al., 2015; Serino, Noel, et al., 2015), we defined the multisensory facilitation as the difference between multisensory (visuotactile) and unisensory (tactile only) reaction times, and performed our analyses on this quantity. The multisensory facilitation was computed by averaging unisensory trials for each subject and experimental block and subtracting it from each visuotactile RT.

RTs were measured in a 2 × 3 design with the factors Hand position (Left, Right), and Trajectory, indicating the direction of the ball, (Left, Right, Receding). Trajectory was recoded as Congruency: Congruent, when the ball was moving towards the tactilely stimulated hand, Incongruent, when it was moving towards the opposite side, and Receding. Reaction times were then analysed by means of linear mixed‐effects models. We fit a model on to multisensory facilitation (MF), including Congruency and Position as predictors. Different random structures were tested, assessing all the five possible combinations of Position and Congruency, including their interaction and just a random intercept, and the model giving the best fit was selected. Both in terms of Akaike and Bayesian Information Criterion, the best model was that which considered only the position as a random factor:

$$\text{MF}∼\text{Position}+\text{Congruency}+\left(\text{Position}|\text{Subject}\right).$$

Additionally, a model including a Position*Congruency interaction was tested, and statistical testing confirmed the selected random structure. Data preprocessing and further analysis was run in R (R version 3.4.4, for linear mixed‐effects model: packages lme4 version 1.1‐15 and lmerTest version 2.0‐36). Linear mixed‐effects models were tested using the Satterthwaite approximation for the degrees of freedom from the lmerTest package.

## 3 RESULTS

### 3.1 Learned connectivity and receptive fields

**FIGURE 2:**
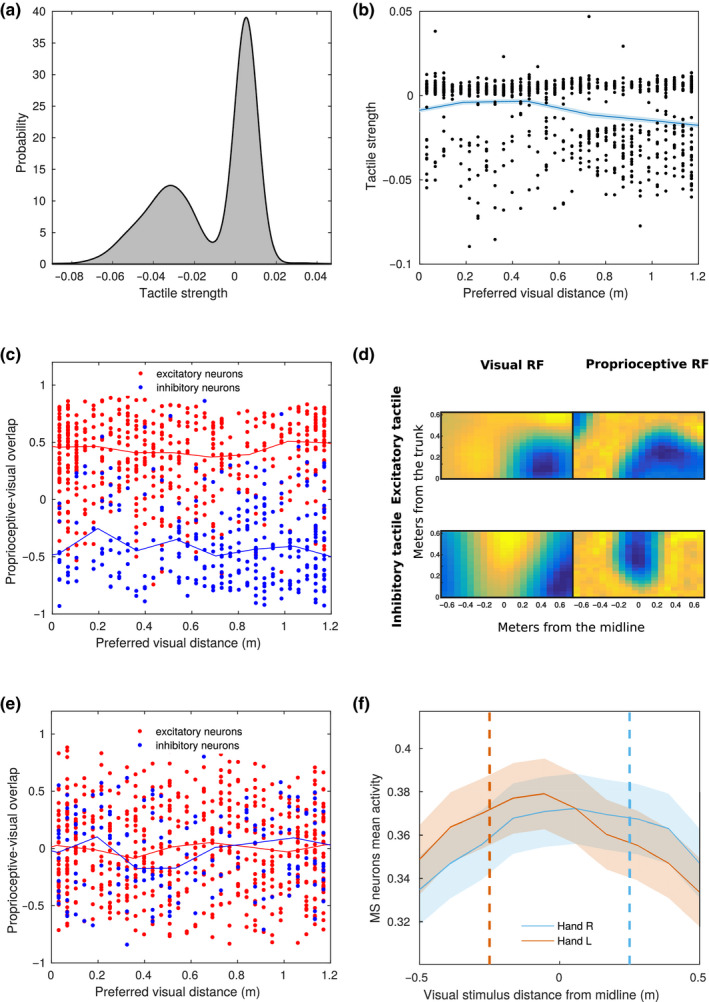
Properties of neurons in the upper layer. (a) Distribution of the strength of tactile input across the multisensory neurons. The strength of the input for each multisensory neuron is defined as the average of the synaptic weight of the projections it receives from the 30 tactile neurons. (b) Dependence of the strength of tactile input on the preferred visual distance of the multisensory neurons. The overlaid solid line represents mean values over 10 distance bins and the shade its standard error. (c) Quantification of the overlap of proprioceptive and visual receptive fields as a function of the preferred visual distance. The overlap is defined as the Pearson correlation coefficient of synaptic input to the multisensory neuron over space. Red and blue denote respectively multisensory neurons projecting excitatory and inhibitory synapses towards the tactile area. The overlaid solid lines represent mean values over 10 bins, with the shade representing the standard error. (d) Two exemplary visual (left) and proprioceptive (right) receptive fields of multisensory neurons. in the upper panels, a neuron receiving and sending excitatory projections to the tactile area, with overlapping visual and proprioceptive RFs. In the lower panels, a neuron receiving and sending inhibitory projections to the tactile area, with disjoint visual and proprioceptive RFs. Yellow and blue indicate respectively strong and weak projections from the unisensory areas to the multisensory neurons. (e) Same as panel c, but in a control model where tactile input was provided randomly and uncorrelated with visual and proprioceptive information. (f) Mean activity of the multisensory neurons that positively respond to touch, as a function of the position of the visual stimulus. The orange and light blue curves correspond to two different simulated positions of the hand, respectively, 25 cm left and right of the midline

The neural network was designed to learn a connectivity scheme that optimizes the reconstruction of the patterns of stimulation observed during the training. This led to a spontaneous diversification of the response of neurons in the multisensory layer to the different sensory modalities. Since at initialization all the multisensory neurons are connected with all the neurons in unisensory populations, after the training all neurons were to some extent multisensory, meaning that they received some input from all the unisensory populations. Nevertheless, a great variability in the modality tuning of different neurons and thus in the pattern of sensory responses emerged. In order to quantify the response of each neuron to a given modality, we computed the sum of the absolute value of the strength of synapses from a given unisensory population, normalized by the mean input for that modality. In particular, we focused on the response of multisensory neurons to tactile inputs. As appears evident from Figure 2a, the response has a bimodal profile, meaning that the population spontaneously diversifies in inhibitory and excitatory neurons as a function of tactile inputs. In general, around 55% of the neurons were found to receive excitatory projections from the tactile area, with the remaining 45% receiving inhibitory projections.

In order to test whether and how the model might build up a PPS representation from capturing regularities in the environment, we tested how the spatial properties of the multisensory neurons depended on their tuning to the tactile modality. It is known from neurophysiological literature that the PPS is represented in the monkey cortex by a set of multisensory neurons that respond both to touch on a given body part, and to visual stimuli close to that body part. In the previous version of the model, this evidence was implemented by a hard‐wired connectivity whereby the projections to the multisensory neuron(s) were of fixed strength from the tactile area, whereas from the visual area they decreased as a function of the distance from the hand. We asked whether our model could simply learn a similar pattern of connectivity from the multisensory training, in which neurons that respond more strongly to touch code mostly for the close (trunk‐centred) visual space. Since our model uses several multisensory neurons, that spontaneously tune differently to each sensory modality, we tried to define a suitable approach to test this hypothesis. For each multisensory neuron, we defined its preferred visual distance as the preferred distance (along the anterior‐posterior axis) of the visual unisensory neuron that projects the strongest excitatory synapse to that same multisensory neuron. Roughly, this corresponds to the peak of the visual RF of the multisensory neuron. This allowed us to explore how the properties of multisensory neurons vary depending on the region of the visual space that is stimulated. We found that, on average, the tactile input computed by multisensory neurons slightly decreases with their preferred visual distance, coded as described above (Figure 2b). Excitatory neurons tend have the peak of their visual RF close to the trunk while inhibitory neurons tend to have it in the far space. This goes in the same direction as the synaptic connectivity in the previous neural network model, but here the distance dependent modulation is much weaker, and does not clearly differentiate the close and the far space. This may seem surprising, but due to the width and complex shape of the RFs learned by most multisensory neurons, the visual preferred distance of a given multisensory neuron is not always informative. A multisensory neuron with the peak of its receptive field in the far space can still have a significant response to close stimuli, and vice versa. More importantly, since in our architecture the visual input was not coded in hand‐centred coordinates, the presence of tactile input does not simply depend on the distance in the visual space, but on proprioceptive and visual information combined. Likely, the slight dependence of connectivity on distance is mainly explained by the fact that the proprioceptive hand position cannot be further than 60 cm away from the trunk. However, the presence of the proprioceptive population introduced an additional level of complexity in the neural network, that can be appropriately addressed only by looking at the associations learned by the network between the visual and the proprioceptive coding. To do this, we computed the overlap between proprioceptive and visual receptive fields of each multisensory neuron, defined as the spatial correlation of its incoming visual and proprioceptive synaptic weights. This quantity approximatively corresponds to the spatial correlation of its visual and proprioceptive RFs. Since the spacing in the grid of neurons is different for the two populations, the correlation was computed after interpolating the proprioceptive synaptic weights on a grid of points with the same spacing of the visual population. A positive overlap means that the neuron tends to be activated when the hand and the visual stimulus are in the same position, whereas a negative overlap indicates that the neuron responds when the hand and the stimulus are far away. We expected the nature of the learned visuo‐proprioceptive associations of a given multisensory neuron to depend on its response to tactile input, therefore we divided the heterogeneous population of multisensory neurons in two groups, based on whether they are inhibited or excited by tactile inputs. Then, we studied the dependence of such overlap on the preferred visual distance. For excitatory neurons, the visuo‐proprioceptive overlap was strong regardless of their preferred visual distance. Conversely, for inhibitory neurons, the overlap was strongly negative at all preferred visual distances (Figure 2c). An exemplary pair of inhibitory and excitatory neurons with preferred distance in the close space are shown in Figure 2d. These results suggest that, more than differentiating between neurons coding for the close and the far space overall, the network spontaneously organized them in two populations of overlapping and anti‐overlapping visual and proprioceptive RFs. Again, due to the width and complex shape of RFs, the presence of neurons with strong visuo‐proprioceptive overlap, and preferred visual distance in the far space should not surprise. Crucially, in the present model, the alignment (or anti‐alignment) of receptive fields emerges from the statistical regularity of touch with respect to an external visual stimulus and proprioceptive information. In order to demonstrate this, we replicated the simulation represented in Figure 2c after a control training with the same visual and proprioceptive stimuli, but in which touch was provided randomly and independently from the hand‐centred coordinates of the visual stimulus. The visuo‐proprioceptive overlap was always close to zero for both excitatory and inhibitory neurons (Figure 2e). In order to establish a comparison with the neurophysiological literature, we then studied the subset of neurons in the multisensory layer that positively respond to touch, to compare our artificial neural population to the one typically studied in primates (Fogassi et al., 1996; Graziano et al., 1994, 1997). In Figure 2f we show the average response of such neurons as a function of the position of the visual stimulus, in two conditions: hand to the left and to the right of the body midline. The average receptive field of the population shifts according to the hand position, in a similar way to what was reported by Graziano for individual neurons (Graziano et al., 1997).

### 3.2 The network encodes tactile predictions in hand‐centred coordinates

In an RBM, information from the different unisensory populations of the lower layer is encoded in the upper layer in a unified and compressed representation, embedding the statistical relations between the unisensory inputs. This allows the network to build a more compact and accurate representation of the input than each of its unisensory components (Makin et al., 2013), and can be seen as a predictive form of multisensory integration, in which inputs from different modalities influence and complement each other to better fit a global model of sensory inputs. We hypothesize that PPS representation spontaneously emerges when a neural network learns to integrate in such a way external and body‐related information, being trained on sensory inputs that reflect the natural statistics of body‐environment interactions. In practice, to efficiently encode incoming sensory information, multisensory neurons in our network must learn an encoding schema that embeds the statistical relations observed between tactile stimulation on the hand and visual stimuli close to the hand (the hand position being specified via proprioceptive information). As a consequence, when a visual stimulus is present close to the proprioceptively encoded hand position, we expect the multisensory neurons to start coding for the presence of tactile stimulation at a sub‐threshold level, before or even in absence of contact. This prediction constitutes a possible explanation of the well‐reported effect of a facilitation of reaction time to tactile stimulation in the presence of an external stimulus approaching the stimulated body part (Canzoneri et al., 2012; Serino, 2019). Indeed, the “pre‐encoding” of tactile information in multisensory neurons might be not sufficient to elicit conscious tactile perception, but might boost responsiveness to tactile stimulation, thus speeding up reaction times when tactile stimulation is delivered. Following this line of reasoning, testing our hypothesis becomes equivalent to performing in‐silico simulations of tactile detection tasks such as in (Canzoneri et al., 2012). Practically, this can be done by providing proprioceptive and visual information to the neural network, while suppressing the input from the tactile area, so as to measure only the contribution of vision and proprioception on the tactile information encoded in the multisensory layer. The activity we read out from the tactile population (even in absence of tactile stimulation) is used as a proxy of multisensory facilitation, i.e., faster reaction times in a tactile detection task. We call this read‐out tactile information evoked tactile activity, and treat it as a in silico behavioural correlate of PPS representation. Note that this does not necessarily mean that behavioural effects in reaction times reduction are linked to actual activity in tactile unisensory areas, as behaviour may be based on the amount of tactile information contained in the multisensory layer, that we only decode through feedback synapses. Consistently with our previous theoretical reasoning and with neurophysiological and behavioural findings, we expect the evoked tactile activity to depend strongly on the location of the external stimulus with respect to the hand position, i.e., on its hand‐centred coordinates.

**FIGURE 3:**
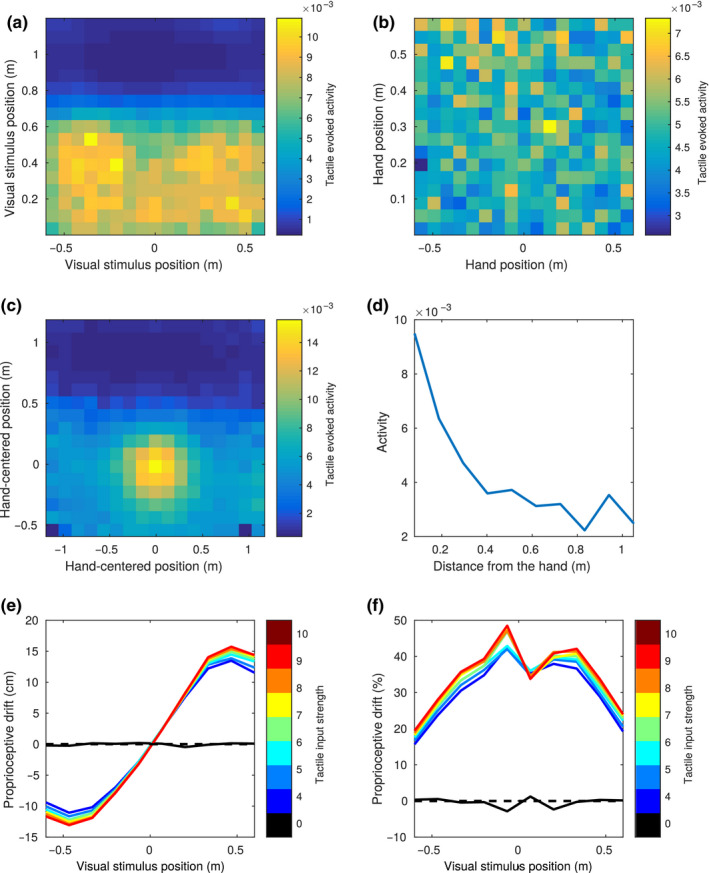
Simulated behavioural experiments. (a and b) Tactile evoked activity ‐ multisensory facilitation as a function of visual stimulus position (in trunk‐centred coordinates) and hand position. The evoked tactile activity is obtained by setting the tactile input to zero, encoding a visual and a proprioceptive input, and reading out the tactile information encoded in the multisensory area from the tactile area (i.e.: its mean activity after a “down” pass). In trunk‐centred coordinates (a) stronger activity for close positions of the visual stimulus can be observed, but no modulation as a function of the position along the anterior‐posterior axis. Virtually no modulation is observed as a function of hand position (b). (c) The same tactile evoked activity, plotted as a function of the visual stimulus position in hand‐centred coordinates. (d) Tactile evoked activity as a function of the distance from the centre of the hand of the visual stimulus. (e) Simulated proprioceptive drift in the invisible hand illusion. The proprioceptive input is fixed at the midline, and the position of the visual stimulus is shifted across the midline. The plot shows the proprioceptive position reconstructed by the network after integrating the three sensory inputs. The *x* axis represents the distance from the midline of the visual stimulus. Different colours represent different levels of intensity for the tactile input, starting from black (no touch/asynchronous stimulation), to red (maximal intensity of tactile stimulation). (f) Same as panel d, but the proprioceptive drift is expressed as the percentage of the distance between the visual and proprioceptive stimuli

To test this hypothesis, we ran a simulation in which the hand and the visual stimulus were placed at random positions within the respective areas and measured the evoked tactile activity as a function of the position of the hand and of the visual stimulus. As expected, the region of space in which visual stimuli elicit an evoked activity in tactile neurons was spatially anchored to the hand. In particular, if the evoked tactile activity was displayed as a function of hand position or visual position of the stimulus in trunk‐centred coordinates (Figure 3a,b), there was a weak, non‐coherent modulation of activity. Instead, if the activity was displayed as a function of position of the visual stimulus with respect to the hand position as coded by the proprioceptive population, i.e., hand‐centred, (Figure 3c), the modulation became stronger and coherent, with a maximal level of activity when the stimulus was in the origin (i.e. the centre of the hand), sharply decreasing with distance. Figure 3d shows the trend of tactile evoked activity as a function of the distance of the visual stimulus from the centre of the hand. This curve shows a similar trend to what reported for some neurons mapping the PPS representation around the monkey face (Graziano et al., 1997).

### 3.3 In‐silico results match in‐vivo hand‐centred coding of multisensory facilitation

**FIGURE 4:**
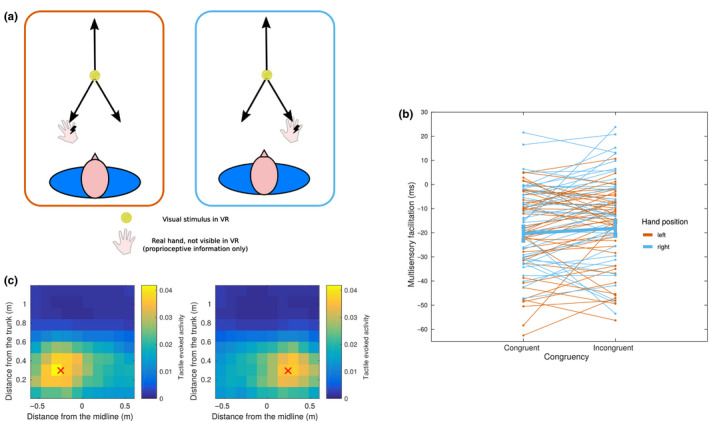
Results of the behavioural experiment. (a) Schematic experimental setup. The subjects placed their right hand approximatively 30 cm in front of their trunk, either 25 cm left or right of their midline. The origin of the arrows represents the starting point of the different trajectories, coinciding with the fixation cross. The total length of the trajectories was approximatively 50 cm. (b) Modulation of average reaction times for the 43 participants as a function of hand position and ball trajectory congruency with hand position. For simplicity, we show only the two conditions that are relevant for confirming our hypothesis, and leave out the receding condition. Thick lines indicate global means by condition. (c) Expected results from model simulations for the same experimental setup. Red crosses represent the position of the real hand's centre, the colour coding represents the predicted multisensory facilitation. Yellow areas represent zones of higher facilitation/faster reaction times

In order to confirm that the proposed architecture can model actual behaviour in a meaningful way, we ran an ad‐hoc behavioural experiment on healthy participants. The aim of the experiment was to show that proprioceptive information is integrated with information about an incoming visual stimulus, affecting tactile processing on the hand. As a behavioural proxy of multisensory integration, we measured reaction times to tactile stimulation on the right hand, while the subjects were seeing task‐irrelevant visual stimuli (tennis balls) in virtual reality. RTs were compared for Hand position (Left, Right) and Congruency of the ball trajectory (Congruent, Incongruent and Receding). A linear mixed‐effects model on the multisensory facilitation (MF), including Congruency and Position as predictors (see Methods for details), showed a significant main effect of Congruency (*F*(2, 4,831.8) = 6.389, *p* = 0.0017) and a marginally significant effect of Position (*F*(1, 42.1) = 3.59, *p* = 0.065). When looking at individual coefficients, using the Receding trajectory as a reference, we found Congruent trials to be significantly faster (−4.115 ms, *SE* = 1.184 ms, *T* = −3.477, *p* < 0.001), and Incongruent trials to be not significantly different (−1.216 ms, *SE* = 1.188 ms, *T* = −1.024, *p* = 0.30) from receding trials. In order to directly compare Congruent and Incongruent trials, and assess the role of proprioception in visuotactile integration, we fit the same model on the subset of Congruent and Incongruent trials. Again, the main effect of Congruency was significant (*F*(2, 3,202.4) = 5.912, *p* = 0.015), with Congruent trials faster than Incongruent trials (−2.889 ms, *SE* = 1.188 ms, *T* = −2.432, *p* = 0.015). This is in line with the model's qualitative predictions, shown in Figure 4c. Additionally, to rule out the possibility that the congruency effect may be present only on one side of the midline, we run the same model including a Position*Congruency interaction. This did not change the main effect of Congruency (*F*(2, 4,831.8) = 6.389, *p* = 0.0017), nor the comparison Congruent versus Incongruent( *F*(1, 3,201.3) = 5.90, *p* = 0.015), and the interaction was not significant (*p* = 0.76). Note that the facilitation when the visual stimulus is on the opposite side of the midline (Incongruent trials) is close to zero, similarly to when the visual stimulus is in the region outside the PPS (Receding trials), in line with the non‐significant difference found in our experiments. It is worth noting that the multisensory facilitation compared to unisensory trials was significantly below zero in all conditions, including Receding trials (−14.6 ms, *SE* = 2.45 ms, *T* = −5.96, *p* < 0.001). This seems to contradict model predictions, as no significant tactile evoked activity is expected in the far space. We hypothesize that this may be due to overall stronger expectation effects in multisensory trials, compared to unisensory trials, due to the presence of the virtual ball providing a more precise cue about the likely time of stimulation. Our experimental design, unlike most previous studies, makes the delay of stimulation orthogonal to the three conditions of interest, which allows comparing them while controlling for expectation. We also investigated more in detail the possible interactions between PPS representation and expectation effects, by analysing trials separately by stimulation delay. We found an overall effect of stimulation delay reducing reaction times, compatibly with the presence of expectation effects. However, the decrease in reaction times with increasing delay (and decreasing distance from the hand) was significantly stronger in the Congruent condition, further confirming the presence of proximity effects in modulating reaction times (see Supporting Information and Figure S4 for details).

### 3.4 The network encodes proprioceptive inputs as a function of visuotactile integration – The IHI

Our previous computational (and behavioural) results show how visual and proprioceptive information combined can affect the encoding of tactile information to reproduce the associations learned during the training (or real‐life experience). Since the learned associations have no preferential direction, we expect the transfer of information between sensory modalities to take place also in the opposite direction: from the tactile to the visual and proprioceptive modalities. In particular, we focused on how visuotactile inputs affect the encoded proprioceptive information, as this link has been previously investigated in several behavioural works exploring the multisensory bases of body representation (Guterstam et al., 2013; Salomon et al., 2017). We fixed the input hand position at the midline and provided visual stimulation at different positions along the anterior‐posterior axis. This was done in association with no tactile inputs (touch OFF), or at various levels of intensity of tactile stimulation (touch ON). We can consider the “touch ON” conditions as synchronous stimulation, in which touch and visual stimulation occurred at the same time, and the “touch OFF” as asynchronous stimulation, meaning that visual stimulation and touch were sufficiently separated in time to have no residual activity in the tactile area when visual stimulation occurred. Then, as we previously did with the tactile population, we projected the multisensory activity to the proprioceptive population, and computed the integrated proprioceptive position as the barycentre of neural activity. In the “touch ON‐synchronous” condition, we found that the proprioceptively encoded position of the hand gets attracted towards the position of the visual stimulation. This result held with little changes at different intensities of tactile stimulation, as if the presence of tactile stimulation was treated as an all or none variable to generate the attractive pull (Figure 3e,f). Only at zero tactile intensity, in the “touch OFF‐asynchronous” condition, was the reconstructed proprioceptive position roughly unbiased and did it correspond to the actual proprioceptively encoded hand position. These results resemble behavioural findings reported by Guterstam et al. (2013) when introducing the so‐called “IHI.” In the IHI, the hand of a participant is hidden, and tactile stimulation is provided while synchronously stroking the empty space next to the location of the real hand. Thus, as in our model, the subjects receive visual information about an external stimulus, touch on the hand, while processing proprioceptive cues, while they do not get any visual information about the hand position. Participants report feeling to have an “invisible hand” and when asked to point at the location of their real hand, they aim to a location shifted towards the point in space where the visual stroking occurred, a phenomenon known as proprioceptive drift. The output of the proprioceptive population in our model simulation in the “touch‐ON” condition replicates proprioceptive drift in the IHI.

### 3.5 Development of the key features of the network during training

**FIGURE 5:**
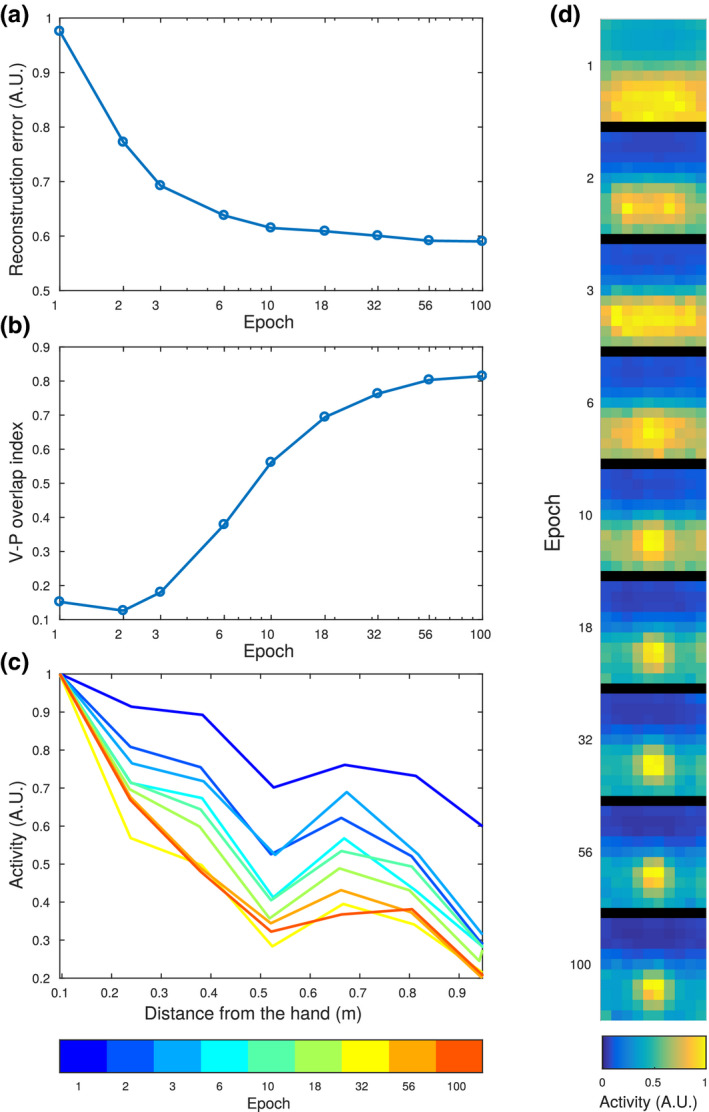
Evolution of the network during training. (a) Reconstruction error of the network plotted as a function of the training epoch. The reconstruction error is defined as the mean squared difference between the training sensory input and its reconstruction in the confabulation phase. (b) Visuo‐proprioceptive overlap index across the 9 training epochs. The visuo‐proprioceptive overlap index is defined as the difference between the average visuo‐proprioceptive overlap of tactile excitatory and tactile inhibitory neurons. The stronger the overlap for tactile excitatory neurons, and the stronger the anti‐overlap for tactile inhibitory neurons, the higher the index is. (c) Evoked tactile activity as a function of the distance from the hand of the visual stimulus, across the same nine epochs of training. (d) Evoked tactile activity as a function of the position of the stimulus expressed in hand centred coordinates. The activity is plotted for the same nine stages of training

After outlining the main features of the network, we explored how, during the training, these develop from the initial random connectivity. The results are summarized in Figure 5. To simply quantify the overall progress in the training of the network we computed the reconstruction error. This quantity is defined by encoding a sensory input in the multisensory layer and then projecting it back to the unisensory areas. The mean squared difference between the original input and the reconstructed activity is called reconstruction error, and it is expected to decrease during the training as the network learns to more efficiently encode its sensory inputs. As the training progressed, the reconstruction error decreased (Figure 5a), meaning that the network learned to reproduce more reliably the information contained in the unisensory inputs, after encoding it in the multisensory layer. After the initial strong decrease of the reconstruction error (from epoch 1 to epoch 6), the learning slowed down, and continued at a reduced pace throughout the whole training, probably towards the saturation value due to the stochasticity of the network's update rule. In order to synthesize the information about the overlap of visual and proprioceptive receptive fields, and display its evolution across epochs, we define a visuo‐proprioceptive overlap index. The visuo‐proprioceptive overlap index is defined as the difference between the average visuo‐proprioceptive overlap of tactile excitatory and tactile inhibitory neurons. At the beginning of the training, the overlap index was low and close to zero, meaning that inhibitory and excitatory tactile neurons are not differentiated in terms of visual and proprioceptive RFs. During training, the value progressively increased, reaching almost the final value after epoch 18 (Figure 5b). This seems to coincide with the emergence of a strong tuning of the tactile evoked response to the distance from the hand (Figure 5c). As seen in Figure 5d, in the first stages of training, the reconstructed tactile activity was coarsely determined by the distance from the body of the visual stimuli. At this stage, the network has only learned that touch is more likely to occur if a visual stimulus is in the closed space, and still does not take proprioceptive information into account. Starting from epoch 10, and more clearly from epoch 18 and onwards, the network's response became tuned to hand‐centred coordinates, as determined by proprioceptive signals.

### 3.6 Visually encoded hand position

**FIGURE 6:**
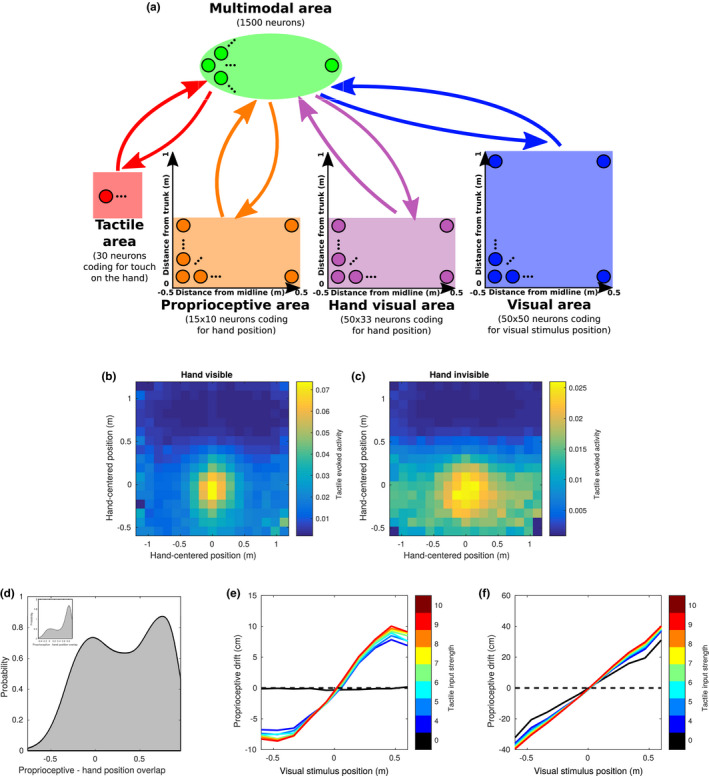
Network including visual information about hand position (a) Architecture of the network. In addition to the previous model, this network has one visual population (purple one) coding for the position of the hand. The tuning curves of neurons in this population have the same width as in the visual population coding for the position of the external stimulus. Other populations' tuning curves and training parameters were the same as in the previous model. (b) Tactile evoked response as a function of the position of the stimulus expressed in hand centred coordinates. (c) Same as panel (b), but the activity in the visual population coding for the hand was set to 0, simulating the occlusion of the hand and reproducing the sensory input of the previous model. (d) Distribution of the overlap between proprioceptive receptive fields and the receptive fields of the visual population coding for hand position. The inset shows the same result, in a network in which the proprioceptive and visual hand positions were never dissociated. (e) Proprioceptive drift in the simulated invisible hand illusion. We followed the same procedure as for Figure 3e, and set the activity in the visual population coding for hand position to 0 to simulate the occlusion of the hand in this network. The x axis represents the distance from the midline of the visual stimulus. (f) Proprioceptive drift in the simulated rubber hand illusion. The procedure was the same as for the invisible hand illusion, with the exception that the visual hand area was now coding for the same location as the external visual stimulus

In the present work, we limited the inputs about hand position to proprioceptive information. This was done mainly to minimize the network's complexity and the number of input populations, facilitating the task of reverse engineering the network's functioning. Nevertheless, it is known from neurophysiological literature that visual input about arm (or even artificial reproductions of the arm) position affects the response of some PPS neurons (Graziano, 2000). However, since proprioceptive and visual information are redundant, at least in normal conditions, we predicted that adding visual cues about the hand position would not affect significantly the main properties of the network. To show this, we trained another network identical to the one shown in the previous paragraphs, with the addition of another visual population, coding for the location of the hand in space, through the same population coding and tuning curves used for the external visual stimulus (Figure 6a). In 75% of the training examples, the additional visual population coded for the same position in space as the proprioceptive population. In addition, to model occlusion of the hand by other objects or its exclusion from the visual field, we suppressed visual information about hand position in 25% of the training examples. To model the vision of other people's hands, the visually and proprioceptively encoded positions of the hand were independent in 25% of the trials. Therefore, we modelled the hand visual area as a neural population coding for the spatial location of hand‐like objects in space, without recognizing the specific visual features of one's own hand. Then, we run the same set of analyses as in the previous paragraphs. In Figure 6b,c, we show how the network encodes information in hand‐centred coordinates, similarly to what shown in Figure 3b. We tested the network both in the case of visible (Figure 6b) and invisible (occluded) hand (Figure 6c) and found comparable results, the only difference being a weaker evoked activation of the tactile area when the hand was not visible. Even when the network was trained with both visual and proprioceptive information, proprioception alone was sufficient to build a visuotactile PPS representation. In Figure 6d we provide a simple explanation for this: in the majority of multisensory neurons, the learned proprioceptive and hand‐visual receptive fields were strongly overlapping, as the two populations typically code for the same spatial location. Interestingly, the visuo‐proprioceptive overlap distribution in Figure 6d presents a secondary peak at zero overlap, besides the main peak around 0.75, showing that the receptive fields were completely dissociated in a minor yet significant fraction of the multisensory neurons. Further testing showed that this was the case only when the network had been exposed to the dissociated visual and proprioceptive hand positions (others' hands) during training. When training an identical network, in which visual and proprioceptive hand positions were always overlapping, the zero overlap peak was greatly reduced, as seen in the inset of Figure 6d. This may reflect the network learning to differentiate between integration and segregation of visuo‐proprioceptive information (see Section 4). We then tested the IHI, by providing the same inputs as previously done for Figure 3e,f in the visual, tactile and proprioceptive populations, and no input in the hand visual population. The results closely matched the ones of the previous model (Figure 6e). Moreover, this extended network architecture reproduced the stimulation pattern of the rubber hand illusion. We fixed the proprioceptive hand position, while encoding an incongruent hand position in the visual hand area, representing the rubber hand. At the same time, we provided visual stimuli at the same location as the visual hand, representing the stimulating brush, and either no tactile stimulation or touch at various intensities. Then, we read out the proprioceptive hand position, by projecting multisensory activity down to the proprioceptive population. We observed a significant proprioceptive drift towards the rubber hand in the touch ON‐synchronous that was weakly modulated by tactile intensity (Figure 6f). A significant proprioceptive drift was observed also in the touch OFF‐asynchronous condition, although clearly smaller than in the synchronous condition. This result, seemly surprising, is actually in line with behavioural reports of a significant proprioceptive drift towards the rubber hand even in the case of no or asynchronous visual stimulation (Rohde et al., 2011; Samad et al., 2015).

### 3.7 Shifting receptive fields at the level of single neurons

**FIGURE 7:**
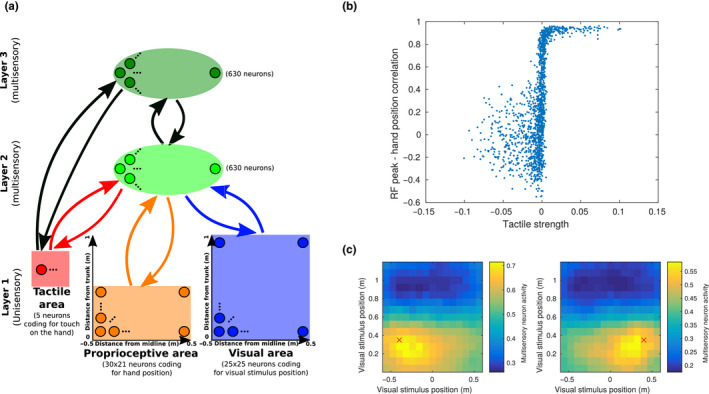
Individually shifting receptive fields. (a) Architecture of the network. The first two layers have the same architecture as in the main model, but fewer neurons to facilitate the training. The third layer is connected to the second multisensory layer and to the tactile population in the unisensory layer. The training was performed in two steps. The first step was identical to the original model. In the second step, training inputs for the second multisensory layer were constituted by the joint activity of first multisensory layer neurons and unisensory tactile neurons. (b) Correlation between hand position and RF peak of second multisensory layer neurons, as a function of the strength of the input they receive from unisensory tactile neurons. The correlation is defined as the average between correlations along the *x* and *y* directions. (c) Visual receptive field of one exemplary multisensory neuron in the third layer, receiving strong excitatory projections from the tactile area, for two different hand positions. Each subplot corresponds to different position of the hand, indicated by the red cross overlaid to the receptive field

In the previous paragraphs, we showed how the network can encode information in hand‐centred coordinates at the population level. This allowed to reproduce some important behavioural and neurophysiological aspects of PPS representation. However, while neurophysiological studies reported individual neurons with visual receptive fields spatially anchored to body parts in space (Graziano, 1999, 2000), the receptive fields of individual multisensory neurons in our network cannot be spatially “shifted” by proprioceptive inputs. Mathematically, this is a direct consequence of the fact that the network has only two layers, and that the response of one multisensory neuron is a sigmoidal function of the sum of its inputs, with the visual and proprioceptive inputs being independent. Since the sigmoid is a monotonically increasing function, when changing the proprioceptively encoded hand position, the neuron's response as a function of the visual stimulus' position would either increase or decrease everywhere, but do not change its global spatial properties. More specifically, the peak of the receptive field would not change. However, since the two‐layers network learned to encode information in hand‐centred coordinates at the population level, we expect that the addition of a third multisensory layer could lead to individual neurons with visual receptive fields anchored to body parts in space. We therefore trained a further model to provide an example of how fully hand‐centred receptive fields at the single neuron level can be achieved by simply expanding our two‐layers architecture. The new network had the same architecture as in our previous model, but with reduced overall number of neurons, to keep its computational complexity manageable during the learning task. We then added a second, “higher level,” multisensory layer, receiving inputs from the first multisensory layer and from the tactile area (Figure 7a). The training was performed in two steps. In the first step, connections between unisensory areas and the first multisensory layer were trained as shown before, with contrastive divergence and coupled unisensory inputs (Figure 1b). After completion of the first step of training, connections from the tactile area and the first multisensory layer to the second multisensory layer (denoted by black arrows in Figure 7a) were again trained with contrastive divergence. The stimuli were generated by encoding unisensory inputs from the usual training set in the first multisensory layer and using the so obtained activity, coupled with activity in the tactile area, as training input for the second multisensory layer. The hypothesis underlying the emergence of shifting RFs from this architecture stems from our previous observation that multisensory neurons that respond to touch have overlapping visual and proprioceptive receptive fields. We expect individual neurons in the second multisensory layer to learn the associations between (unisensory) tactile activity and the activity of neurons in the first multisensory layer that code for touch and whose visual and proprioceptive receptive fields overlap. If this is the case, third‐layer neurons would learn to be active when *any* of the neurons coding for touch in the first multisensory layer is active. That is, they would respond whenever vision and proprioception are aligned, by shifting the peak of their visual receptive field. We therefore expected that the neurons receiving the strongest projections from tactile units would exhibit a stronger tuning to hand position. We explored this hypothesis by setting tactile inputs to zero, and mapping the peak of the RF for 100 different hand positions. We then computed the average correlation (along the *x* and *y* axis) between hand position and RF peak, as an index of hand position tuning. As shown in Figure 7b, neurons receiving the strongest projections from unisensory tactile neurons show the highest degree of hand position tuning, with 27.5% of them having an average correlation coefficient above 0.6. An example of a hand position tuned neuron can be seen in Figure 7c.

### 3.8 Encoding proprioceptive input in joint angles

In the previously presented models, we chose to encode proprioceptive input as a population coding in Cartesian space. This was done to simplify the interpretation and visualization of the results. However, the encoding of raw proprioceptive input likely resembles joint angles more than Cartesian space. Here, we demonstrate how the main results of our work can be recovered when training a network with proprioceptive inputs encoded under the form of more biologically realistic joint angles. In this version of the network, the proprioceptive population was still 15 × 10 neurons, representing, respectively, the angle of the shoulder in the horizontal plane and the angle of the elbow. Shoulder angles ranged from −π/4 to π/2, where 0 represents the arm straight ahead and negative and positive angles represent a deviation towards the body midline or away from it, respectively. Elbow angles ranged from −π/2 to 0, where 0 represents the arm fully extended. Visual and tactile inputs were encoded through a population coding with Gaussian tuning curves, with the same width as in the main network. For training, pairs of proprioceptive and visual positions were drawn from uniform distributions in the respective range (therefore, joint angles were randomly drawn for the arm instead of positions in the Cartesian space). Then, feedforward kinematics were computed to determine hand position in Cartesian coordinates, and tactile units were activated if the distance between the visual stimulus and the hand was smaller than 15 cm.

(9) $$\begin{array}{c} x_{\mathrm{hand}}=0.3\sin\left(\theta_{1}\right)+0.35\sin\left(\theta_{1}+\theta_{2}\right)\\ y_{\mathrm{hand}}=0.3\cos\left(\theta_{1}\right)+0.35\cos\left(\theta_{1}+\theta_{2}\right) \end{array}$$

where 0.3 and 0.35 represent the length of the arm and forearm (up to the centre of the hand), respectively, and *θ*_1_ and *θ*_2_ represent respectively shoulder and elbow angles.

**FIGURE 8:**
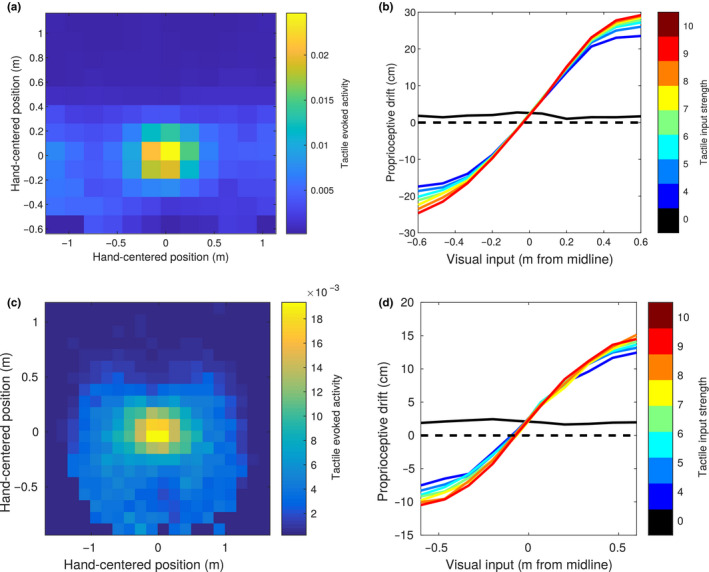
Further network generalizations. Panel (a) shows of the same analyses shown in Figure 3c, for a network in which hand position was encoded under the form of shoulder and elbow joint angles. Panel (b) reproduces Figure 3e for the same network. Panels (c) and (d) demonstrate the same results as panels (a) and (b) in a network where, in addition to encoding proprioceptive inputs under the form of joint angles, a fourth population coding for gaze position was added. This requires the network to compute a further reference frame transformation

Figure 8b,c shows the same results as Figure 3c,e, for this version of the network. The network is able to compute hand‐centred coordinates of visual stimuli in a similar way to what already shown in the main results. Also when testing the proprioceptive drift in the IHI similar results were obtained, except for a slight bias in the reconstructed hand position. This is in line with our expectation that, as long as the network is able to learn a good generative model of its sensory inputs, the specific encoding schema should not matter, provided that information about the physical stimuli can be recovered from the unisensory populations.

### 3.9 Adding gaze angle and further generalizations

Similarly, the network presented previously had to learn a simplified version of the actual reference frame transformations that are necessary to link retinotopic visual input and tactile input through proprioception, as we chose to ignore gaze angle. We therefore explored whether the network, in addition to encoding proprioceptive input in joint angles, could handle the additional degree of freedom of gaze direction in the horizontal plane. We trained another network that was identical to the one presented in the previous paragraph, except for a fourth population coding for gaze direction. This population consisted of 120 neurons, representing gaze angles from −π/4 to π/4, with 0 indicating looking straight ahead, and a tuning curve width of 1 neuron. The small width of the tuning curve, as explained in the methods, is motivated by the necessity to keep the average firing rate approximately constant across the different populations to allow efficient learning. Visual and proprioceptive inputs were encoded as in the previous section, with the difference that the coordinates of the visual input were now eye‐centred. Visual inputs and gaze angles were again uniformly distributed in the respective range, and the body‐centred coordinates of the visual stimulus were determined by rotating its eye‐centred position by the negative gaze angle around the body axis.

(10) $$x_{body‐centred}=R_{z}\left(‐\theta \right)x_{eye‐centred}$$

where *θ* represents the gaze angle, and *R_z_* the rotation matrix along the vertical axis. As usual, tactile stimulation was present if the body‐centred positions of the hand and the visual stimulus differed by less than 15 cm. As summarized in Figure 8c, the network still learned to predict touch in hand‐centred coordinates. We also tested the proprioceptive drift induced in the invisible hand setup (Figure 8d). With proprioception fixed, we measured the proprioceptive drift at different locations of visual stimulation in body‐centred coordinates, but with a random gaze angle (and therefore different retinotopic coordinates) at each trial. The results were again in line with our main findings, with an attractive pull towards the location of visual stimulation but only in the case of tactile stimulation. However, there was a substantial, constant bias also in the case of no‐touch, possibly demonstrating the limits of the network in handling the additional complexity.

## 4 DISCUSSION

### 4.1 Motivation and approach

The multisensory bases of PPS representation have been studied first in animal neurophysiological studies (see Cléry et al., 2015; Graziano & Cooke, 2006) and later in human neuropsychological, behavioural and neuroimaging studies (see Serino, 2019 for a review). Only more recently, efforts have been made to build neural‐network models accounting for the properties of PPS representation in a computational framework (Magosso, Ursino, et al., 2010; Roncone et al., 2016; Straka & Hoffmann, 2017). Shortly after, computational models inspired by visuotactile PPS properties were proposed for impact avoidance (Nguyen et al., 2018), reaching (Juett & Kuipers, 2019) or development of a body schema (Pugach et al., 2019) in robotics. Here we focused on neuroscientific implications of neural network models of PPS representation, by tackling two main questions. First, we asked how the reference frame transformations that are needed to represent visual, proprioceptive and tactile inputs in a common, body‐centred reference frame, could be implemented in a conceptually simple and biologically plausible neural network. We proposed that spatially aligned visual and proprioceptive multisensory receptive fields collectively account for the reference frame transformations that allow the maintenance of the overlap between visual and tactile receptive fields, which is at the core of PPS representation. Second, such alignment of reference frames was obtained through the spontaneous tuning of the synaptic connectivity within the neural network as a function of statistical regularities in the environment. Empirical evidence on the high plasticity of PPS representation (Cléry et al., 2015; Maravita & Iriki, 2004; Serino, 2019) suggests that the synaptic changes due to multisensory stimulation during interactions with the environment play a major role in shaping PPS representation. Here, we argue that the same mechanism can be used to explain how PPS representation is formed at a first stage. Therefore, the learning component is fundamental in a neural network model aimed at describing the key proprieties and the emergence of PPS representation. To achieve these goals, we combined findings and methods from two different approaches applied to model multisensory integration and reference frames transformations. We started from the neural network model developed by Magosso and colleagues (Magosso, Ursino, et al., 2010). The model represents PPS representation as the interaction between unisensory areas processing tactile and visual/auditory information and a multisensory layer, integrating the two unisensory inputs in pre‐computed spatially overlapping receptive fields. We integrated this approach with further computational models of reference frame transformations, proposed by Ma et al. (2006) and Makin et al. (2013). Ma and colleagues were able to generate coordinate transformations in a neural network model using three interconnected populations of neurons with Gaussian receptive fields, whose synaptic weights were hard‐wired. Instead, to model reference frame transformations as learned from sensory inputs, Makin et al. (2013) adapted a neural‐network (RBM) that has been widely used to model complex probability distributions in machine learning. They showed that, indeed, coordinate transformations can be learned from a sensory stimulation based on population coding. Here, we applied the same principles to the key set of sensory inputs that we assumed to be sufficient to build a PPS representation, by implementing an RBM in the architecture proposed by Magosso, Ursino, et al. (2010). In addition to unisensory tactile and visual populations, a proprioceptive population was added allowing the model to processes information related to the position of body parts in space. Importantly, the synaptic connectivity between the unisensory and the multisensory populations was learned through a biologically plausible learning rule, using a set of ecological stimuli as training inputs.

### 4.2 Visuo‐tactile facilitation in hand‐centred reference frames emerges from statistical regularities in the environment

Following classical behavioural and neurophysiological assessments, we focused on visuotactile interactions, and how they are modulated by proprioception, to test PPS representation as emerging from the network. To this aim, visual and proprioceptive inputs in the multisensory layer were encoded in the network, while tactile input were fixed at zero, and the activity induced in the tactile population (through feedback projections) was measured. Such tactile induced activity can be interpreted as the network's prediction of tactile stimulation, based on the integration of visual and proprioceptive information. We found the network's tactile predictions to be based on the hand‐centred coordinates of the visual stimulus, with a maximal strength when visual stimuli are close to the hand and an activation profile depending on the distance from the hand, closely resembling what reported from single cell responses by neurophysiological studies in monkeys (as in Graziano et al., 1997). This pattern of response can be linked to the well‐known behavioural finding that visual (or auditory) stimuli close to a body part induce a facilitation of tactile processing for the same body part (Canzoneri et al., 2012; Spence et al., 2004). Here, we directly replicated this effect in a behavioural experiment on healthy participants. By suppressing visual information about hand position, which is rarely done in similar behavioural studies, we confirmed the relevance of the proprioceptive‐visual associations (as learned by our model) for multisensory integration in the PPS. Our new behavioural data show that tactile responses were facilitated selectively when the side of visual stimulation matched that of the hand position as specified by proprioception. The fact that congruent visual and proprioceptive spatial cues affect multisensory processing is well‐known in experimental psychology, typically shown by the crossmodal congruency effect (Pavani et al., 2000; Spence et al., 2000). However, this had never been demonstrated in a tactile detection task, where the presence of visual cues about hand position is typically thought to be the main driving force. Nevertheless, the comparison between model predictions and behavioural data remains qualitative at the present stage, as the main goal of the experiments presented in this paper was to demonstrate the plausibility of the model's architecture. Further efforts should focus on finding better methods to link model predictions to behavioural data, and increasing the granularity of behavioural measures.

Importantly, the fact that the receptive fields are learned and not hard‐wired allows us to treat their properties as predictions generated by the model, and not assumptions that are set a priori. Specifically, the model predicts the existence of neurons responding to touch, with overlapping visual and proprioceptive RFs, and neurons not responding to touch with dissociated visual an proprioceptive RFs. The collective behaviour of such neurons leads to the encoding of tactile information being influenced by the hand‐centred coordinates of visual stimuli. Their receptive fields are broad and complex in shape, and neurons do not individually encode information in body‐part centred coordinates. This is consistent with what was found in literature in multisensory neurons, displaying broad RFs and only partially shifting reference frames (Avillac et al., 2005). Nevertheless, seminal neurophysiological studies, such as by Graziano and colleagues (Graziano et al., 1999) showed how proprioceptive inputs can shift the visual receptive fields of individual neurons. While in our two layers network fully‐shifting reference frames can emerge only at the population level (Figure 2f), in further simulations we showed how individual neurons with receptive fields anchored to the hand in space can be spontaneously obtained by letting a third layer learn the associations between tactile inputs and the multisensory representation of sensory inputs. With a three‐layers architecture, we therefore showed how neurons with fully and partially shifting RFs may simply be successive levels of information processing. Interestingly, this also implies that canonical PPS neurons may not be needed for generating hand‐centred visuotactile interactions. Importantly, we showed that the presence of tactile stimulation that is coherent with visual and proprioceptive inputs can lead to the alignment of visual and proprioceptive receptive fields in multisensory neurons, constituting a possible explanation for both PPS representation and reference frame transformations. Moreover, we have shown how changing the encoding schema of proprioceptive inputs, the unisensory tuning curves, or even adding an additional reference frame transformation does not change such a finding, thus strengthening the idea that learning of statistical regularities is indeed the key mechanism of the network. A notable exception to such generalizations was, however, the challenge encountered when we attempted to extend the network to a 3D spatial representation. This may be due to computational limitations, but further investigations would be needed to rule out the possibility that this limit may be intrinsic to the network.

### 4.3 Visuo‐tactile integration explains proprioceptive drift

Similarly to what we did with touch, we then tested the effect of visuotactile stimuli on proprioceptive encoding, by providing visual and proprioceptive inputs, and studying the effect of tactile input on the read‐out proprioceptive information. We found that, in the presence of touch, the encoded proprioceptive position got attracted towards the position of the visual stimulus, replicating the proprioceptive drift induced in the IHI. The maximal magnitude of the forecasted shift is around 40% of the visuo‐proprioceptive disparity, in line with behavioural data (Guterstam et al., 2013). By adding to the model another unisensory population, encoding the location of the hand in space as specified by visual information only, we also reproduced a proprioceptive drift as during the RHI. The IHI and RHI have been used to experimentally study body ownership, as a key component of bodily self consciousness (Blanke, 2012). It has been suggested that the multisensory stimulation underlying those illusions rely on the same multisensory principles at the bases of PPS representation (Blanke et al., 2015; Grivaz et al., 2017; Makin et al., 2007). Interestingly in this sense, visuotactile stimulation can induce a subset of PPS neurons to anchor their RFs to dummy hands (Graziano, 2000). Here, we show how the same computational mechanisms that generate the reference frame transformations needed to represent the PPS also can explain the proprioceptive drift in the IHI (or RHI). Clearly, we cannot infer subjective states from neural network simulations. However, it is known that multisensory bodily illusions induce a proprioceptive shift consistent with the model's predictions, and, on the subjective side, alter the sense of body ownership. While it has been argued that proprioceptive drift can occur in the absence of (explicitly assessed) body ownership (Rohde et al., 2011), the amount of drift is known to correlate with the perceived strength of the illusion (Guterstam et al., 2013; Tsakiris & Haggard, 2005). In other words, while it is a distinct neural phenomenon, it seems to participate to the phenomenology of ownership, and it is arguably its only known correlate that can be assessed in a neural network model. Here, we have demonstrated how such correlate of body ownership can emerge on the basis of simple multisensory integration in PPS. Previous mathematical studies proposed Bayesian inference on the incoming sensory information as a mechanism to explain illusory ownership in the rubber hand illusion (Samad et al., 2015). The crucial difference and novelty of the present work is that our results were instead obtained in an artificial neural network with a biologically plausible learning rule. Unlike mathematical models, the network is not designed for (and probably does not achieve) optimal Bayesian inference, but it shares the same underlying probabilistic approach to brain function. The network reproduces behavioural findings by learning a generative model of sensory inputs, capturing subtle and highly non‐linear relations between patterns of neural activity. For example, the effect of touch on the proprioceptive drift was of the “all or none” kind (Figure 3d–f). Such effect, whose finely tuned non‐linearity would be hard to obtain by chance, reflects the fact that, in the training probability distribution, the spatial coherence of visual and proprioceptive inputs only depends on the presence of tactile stimulation, and not on its intensity. Interestingly, the proprioceptive drift decreased when the distance between the hand (defined via proprioception) and the visual stimuli was larger than around 30 cm. This is coherent with the idea that visuotactile interactions occur only within spatially and temporally compatible regions (Holmes & Spence, 2005; Stein et al., 1989), and possibly explains why the RHI and IHI can only take place if the distance between the real and the fake (invisible) hand is limited (Lloyd, 2007). A recent work (Noel, Samad, et al., 2018) found a pattern of spatially decreasing integration of visual and proprioceptive inputs that closely resembles the one found in our simulations. They suggested that the observed behaviour would be in line with a Bayesian causal inference (BCI) model of the world, whose predictions are the weighted average of two alternative sub‐models. In one sub‐model, the two stimuli are assumed to have the same cause, and their positions are integrated in space, whereas in the alternative sub‐model they are treated as separate events. In this perspective, the mathematical counterpart of body ownership would be the weight attributed to the “one‐cause” sub‐model, as already suggested in (Samad et al., 2015). Recent work by Fang et al. (2019) provided neurophysiological support to this proposal. They trained macaques to perform a reaching task, while recording from their premotor cortex in the presence of different levels of disparity between proprioceptive and visual feedback about hand position. As the level of disparity increased, visuo‐proprioceptive integration progressively decreased. In the same study, in a complementary behavioural assessment in humans, the amount of visuo‐proprioceptive integration was demonstrated to correlate with subjective ownership ratings, and was therefore taken as an implicit measure of ownership. They showed that the amount of integration, discriminating between “same cause” versus “different cause” responses, that is arm ownership versus no‐ownership, could be explained by using a BCI model similar to the one used proposed by Noel, Samad, et al. (2018). Single neurons response also followed two patterns: some neurons tended to integrate visuo‐proprioceptive information, suggesting tuning to the “same cause” model, while others tended to segregate them by responding to proprioceptive input only, suggesting tuning to the “separate causes” model. Interestingly, when we included visual information about arm position in the model, we also found two different patterns of responses from neurons in the multisensory layer: one population of neurons with overlapping and another with dissociated visual (coding hand position) and proprioceptive RFs (Figure 6c). Here, we demonstrated how qualitatively similar results can be obtained in a neural network model that shares with Bayesian models the use of a probabilistic framework to describe brain function, but is not tuned for optimality. Similarly, Ursino and colleagues (Ursino et al., 2017) recently showed that a multisensory effect (i.e., the ventriloquism effect), that has been traditionally explained in the framework of Bayesian inference (Alais & Burr, 2004) can emerge from the organization of multisensory receptive fields.

### 4.4 Ownership and embodiment are grounded in a probabilistic model of the physical structure of the body

As we introduced in the previous paragraph, it may be useful to approach the problem beyond the focus of Bayesian optimality, and under a more general perspective. A key function of the brain is to learn the regularities in the probability distribution of its sensory inputs. Those regularities are then exploited to compress inputs in a simpler, more compact representation, retaining the relevant information about their causes in the external world (Attneave, 1954; Barlow, 1961; Simoncelli & Olshausen, 2001). Here, we applied this general principle to a set of sensory inputs – mimicking real‐life natural stimulation – that we assumed to be sufficient for building a PPS representation. We fed simple representations of visual, proprioceptive and tactile inputs to a network designed to fit them to a statistical model of their interdependences. The key to the emergence of such statistical model is the network's biologically plausible plasticity rule: by adjusting synaptic weights until its spontaneous activity resembles the training inputs, the network learns the joint probability distribution of multisensory signals. The statistical relations between such training inputs were not arbitrarily chosen, as they are constrained by the physical structure of the body and its interactions with the environment: touch is always on the body, thus environmental stimuli associated to touch must occur close to the physical body, and their proximity is encoded based on visual and proprioceptive cues. We then showed how, under such limited hypotheses, both PPS representation and the IHI (or the RHI) spontaneously emerge as consequence of a single and unified inference process, where sensory inputs are treated differently depending on their relation to the body. This means that our network complies to some extent with F. de Vignemont's minimal definition of embodiment, arguably the only one that can be applied to a neural network simulation: “E is embodied if and only if some properties of E are processed in the same way as the properties of one's body” (de Vignemont, 2011).

There are other important features that were not directly modelled here, but could be implemented in a model with an architecture similar to ours, designed to learn the probability distribution of its sensory inputs, in order to extend its level of compliance with such definition. For instance, the present model allows to accurately model only simultaneous stimuli. However, the combination between temporal and spatial processing is key for a dynamical model of the body in space, which is deeply linked to PPS representation, as well as for bodily self‐consciousness. Moreover, a perfect generative model, which does not include temporal features, should in principle not fully account for a PPS representation extending beyond the skin, as it would only learn associations between touch and stimuli currently causing it. Again, this could be instead achieved in the general framework of the learning of a complex probability distribution, extending not only in space, but also in time. Interestingly, the idea of PPS representation as a spatio‐temporal prediction system finds empirical support in the observation that it expands when faster stimuli approach (Noel, Blanke, et al., 2018). Straka and Hoffmann (2017) investigated the dynamical properties of visuotactile integration in PPS by coupling an RBM with a feedforward layer undergoing supervised learning. Alternatively, implementing a recurrent dynamics in our RBM would allow to handle the temporal dynamics with more biological realism (see for example Makin et al., 2015). Similarly, the complexity of the visual input could be increased to replace the population coding of pre‐computed positions of objects in space, with more realistic inputs, starting from retinotopic representations to egocentric representations. This way, the visual appearance of the body would be embedded in the training inputs' distribution, possibly allowing explaining why multisensory bodily illusions work less well (or do not work at all) with objects that do not resemble body parts (Tsakiris et al., 2010). Again, the network's conceptual functioning would still hold on the learning of a joint probability distribution, whose variables would be the neural activities of a retinotopic intensity coding. In principle, this framework could be extended to build a neural network that learns a model of all the possible interactions between the body and the environment. We argue that such a process, of which we successfully modelled few key aspects here, might constitute the neurocomputational basis of body representation, and a substrate for the subjective experience of possessing a body, that is felt as one's own, in interaction with the external world.

## CONFLICTS OF INTEREST

The authors declare no conflict of interest.

## AUTHOR CONTRIBUTIONS

TB, EM and AS developed the conceptual framework. TB designed and implemented the neural network, and performed the simulations, behavioural data collection and data analysis. EM and AS provided supervision in data analysis and interpretation of the results. TB drafted the manuscript, all the authors edited and contributed to the critical revisions of the manuscript and read and approved the final version for submission.

### PEER REVIEW

The peer review history for this article is available at https://publons.com/publon/10.1111/ejn.14981

## Supporting information

Supplementary Material

## Data Availability

MATLAB code for training the main network presented in the paper and to reproduce Figures 2 and 3 are available in the following OSF repository: https://osf.io/w6edv/?view_only=135df79adbd84ce79d01111a1663b7de Further MATLAB code, behavioural data and detailed instructions for the replication of other results are freely available from the first author (TB) upon request.
