# Supplementary material for: From statistical regularities in multisensory inputs to peripersonal space representation and body ownership: Insights from a neural network model
Source: Eur J Neurosci. 2020 Oct 12;53(2):611–36. doi: 10.1111/ejn.14981 (PMC7894138; doi:10.1111/ejn.14981)
Supplement: Supplementary file 1 — Supplementary Material [file EJN-53-611-s001.docx]

Supporting information
From statistical regularities in multisensory inputs to peripersonal space representation and body ownership: insights from a neural network model

# Detailed calculation of unisensory precisions

Here we provide the detailed calculation of the localization precision of unisensory inputs, $\sigma_{x}$, assuming a flat prior on their position. When a stimulus located in **x** is encoded in a unisensory population, it generates a conditional distribution of neural activity in the respective unisensory population **u**. The activity of each unisensory neuron $u_{i}$ is drawn from an independent Poisson distribution whose mean is determined by the stimulus location and the neuron’s tuning curve. In our case, we have:

$$P(\text{u}|\text{x})=\prod_{i} P(u_{i}|\text{x})$$

$$P(u_{i}|\text{x})=Pois(g\cdot exp\left\{ \frac{-||\boldsymbol{x}-{\overset{}{\hat{\boldsymbol{x}}}}_{i}|^{2}}{2\sigma_{TC}^{2}} \right\})$$

Where ${\overset{}{\hat{\boldsymbol{x}}}}_{i}$ denotes the i-th neuron’s preferred position, g is the gain of the stimulus, and $\sigma_{TC}$ is the standard deviation of the tuning curve. Note that here we assume all the tuning curves of neurons within each unisensory population to be identical, except for the preferred position. By combining the two expressions we get:

$$\begin{matrix} & P\left( \text{u} | \text{x} \right)\propto\prod_{i} Pois(g\cdot exp\left\{ \frac{-||\text{x}-{\overset{}{\overset{}{\hat{\boldsymbol{x}}}}}_{i}|^{2}}{2\sigma_{TC}^{2}} \right\})= \\ & =\prod_{i} \frac{g}{u_{i}!}exp\left\{ -ge^{\frac{-||\text{x}-{\overset{}{\overset{}{\hat{\boldsymbol{x}}}}}_{i}|^{2}}{2\sigma_{TC}^{2}}} \right\}exp\left\{ -u_{i}\frac{||\text{x}-{\overset{}{\hat{\boldsymbol{x}}}}_{i}|^{2}}{2\sigma_{TC}^{2}} \right\} \\ & =(\prod_{i} \frac{g}{u_{i}!})exp\left\{ -g\sum_{i} e^{\frac{-||\text{x}-{\overset{}{\hat{\boldsymbol{x}}}}_{i}|^{2}}{2\sigma_{TC}^{2}}} \right\}exp\left\{ -\sum_{i} u_{i}\frac{||\text{x}-{\overset{}{\overset{}{\hat{\boldsymbol{x}}}}}_{i}|^{2}}{2\sigma_{TC}^{2}} \right\} \\ & \approx(\prod_{i} \frac{g}{u_{i}!})exp\left\{ g\sqrt{2\pi\sigma_{TC}} \right\}exp\left\{ -\sum_{i} u_{i}\frac{||\text{x}-{\overset{}{\overset{}{\hat{\boldsymbol{x}}}}}_{i}|^{2}}{2\sigma_{TC}^{2}} \right\} \end{matrix}$$

Where the approximation consists in assuming that the sum in the first exponential consists of enough terms to depend weakly on **x**. This is true if the neurons are tiled densely enough that a large number of them contribute to the sum, and the approximated value of the sum can be computed by an integral. As long as this value is constant, it is not needed to compute the posterior variance, which can be obtained by re-writing the exponent of the second term of the expression as follows:

$$\begin{matrix} -\sum_{i} u_{i}\frac{||\text{x}-{\overset{}{\overset{}{\hat{\boldsymbol{x}}}}}_{i}|^{2}}{2\sigma_{TC}^{2}} & \\ =\frac{\sum_{i} u_{i}}{2\sigma_{TC}^{2}}\left[ \text{x}^{2}-2\text{x}\frac{\sum_{i} {\overset{}{\overset{}{\hat{\boldsymbol{x}}}}}_{i}u_{i}}{\sum_{i} u_{i}}+\frac{\sum_{i} {\overset{}{\overset{}{\hat{\boldsymbol{x}}}}}_{i}2u_{i}}{\sum_{i} u_{i}} \right] & \\ =\frac{\sum_{i} u_{i}}{2\sigma_{TC}^{2}}\left[ \text{x}-\frac{\sum_{i} {\overset{}{\overset{}{\hat{\boldsymbol{x}}}}}_{i}u_{i}}{\sum_{i} u_{i}} \right]^{2}+C & \\ =\frac{\sum_{i} u_{i}}{2\sigma_{TC}^{2}}(\text{x}-\text{x}_{b})^{2}+C & \end{matrix}$$

Where C does not depend on the stimulus location **x**, and $\text{x}_{b}=\frac{\sum{\hat{\boldsymbol{x}}}_{i}u_{i}}{\sum u_{i}}$ is the barycenter of neural activity. The posterior is therefore Gaussian, with mean $\text{x}_{b}$ and standard deviation $\sigma_{x}=\sqrt{\frac{\sigma_{TC}^{2}}{\sum u_{i}}}$. The relevant quantity for estimating $\sigma_{x}$ becomes then the total spike count of each sensory input. If the number of active neurons is large enough, the expected value for this quantity can be approximated by an integral

$$E[\sum_{i} u_{i}]=g\sum_{i} e^{-\frac{{\hat{\boldsymbol{x}}}_{i}^{2}}{2\sigma_{TC}^{2}}}\approx g\int e^{-\frac{{\hat{\boldsymbol{x}}}^{2}}{2\sigma_{TC}^{2}}}d\hat{\boldsymbol{x}}=g(2\pi\sigma_{TC}^{2})^{n\text{/}2}$$

Where n is the dimensionality of the physical space of stimulus position (2 in our case), and for simplicity we have considered a stimulus centred in 0, and performed the calculation in units equal to the neuron grid spacing. Therefore, in such units, we have

$$\sigma_{x}=\sqrt{\frac{\sigma_{TC}^{2}}{g2\pi\sigma_{TC}^{2}}}=\sqrt{\frac{1}{g2\pi}}$$

Note that $\sigma_{x}$, in general, depends on $\sigma_{TC}^{2}$, but not in a 2D grid of neurons. Therefore, in our case, the only way to adjust the stimulus precision is by changing the gain or the density of neurons.


Figure S1: To evaluate the goodness of the approximation, we performed simulations by generating 1000 sets of unisensory stimuli in a fixed position, for different values of the gain. Then, we decoded the maximum-likelihood position of the stimulus as the barycentre of the neural population, and estimated its standard deviation along the x axis. The values were compared to the results obtained from eq. (3), after conversion from neuron grid units to physical space units (see Fig. S1). Overall, the approximation was good, even in the more extreme case of the proprioceptive population, where the relatively small number of neurons could have challenged the assumptions of the approximation.

# Effect of the width of unisensory tuning curves on the results

While the multisensory receptive fields in our network were entirely learned from sensory stimulation, unisensory receptive fields were set a priori, and despite being based on neurophysiological knowledge they present a certain degree of arbitrariness. Namely, the width of the Gaussian tuning curves has been determined mainly on technical grounds, to allow efficient training of an RBM. One may therefore wonder to which extent our results depend on the choice of the unisensory tuning curves. Namely, the spatial extent of the hand-centred region in which visual stimuli elicit tactile predictions (the size of the in-silico PPS) may depend on the width of unisensory receptive fields. We therefore trained a series of replicas of our main network, in which we only changed the width of the tuning curves of the unisensory visual and proprioceptive population, and plotted the evoked tactile activity as a function of the distance from the hand. The range of explored widths has a lower limit in that it cannot get much smaller than one neuron in the proprioceptive population, because the stimulus encoding would become extremely irregular, and it cannot get too big as this would require huge safety margins to avoid edge effects. Within this reasonable range, there was virtually no sensitivity to the width of the tuning curve (see Figure S2). Again, this is in line with the idea that the encoding schema should not matter too much, as long as the network is able to learn a good generative model of its inputs.

Figure S2: PPS spatial properties as a function of the width of unisensory tuning curves. We trained 10 replicas of our main network, and multiplied the width of visual and proprioceptive tuning curves by a fixed factor ranging from 0.2 to 2. Here we show the dependence of tactile evoked activity on the distance from the hand of the visual stimulus, as a function of the tuning curve width. The multiplicative factor is colour coded as depicted in the colorbar, with the lowest value (0.2) corresponding to dark blue and the highest value (2) corresponding to red.

# Optimal number of hidden units and precision

Here we illustrate how the number of hidden units influences the precision with which unisensory positions are encoded in the multisensory layer. This analysis was used to determine the number of hidden units to use in our network, aiming at reaching a sufficiently low information loss when passing from the lower to the upper layer, while respecting a biological principle of efficient encoding and keeping computational demands not too high. In order to do so systematically, we trained 20 other replicas of our network, and systematically changed only the number of hidden units from 10 to 3000. We then used the precision with which the position of unisensory stimuli can be recovered, after encoding them in the multisensory layer as a main proxy of information loss. Such precision has a lower bound in the theoretical precision illustrated in section 1, due to noise in unisensory inputs, so when such bound is reached no information loss takes place in the encoding. Practically, this was assessed by generating random positions for visual and proprioceptive stimuli, encoding them (with noise) in the unisensory layer. Then, unisensory activities were projected in the multisensory layer and read out again from the unisensory populations through the usual procedure. However, since we are interested in the information loss in an “up” pass, the read out is done noiselessly, by taking mean values instead of Poisson samples. For our main analysis, we considered results obtained by performing noisy “up” passes, as the efficient encoding principle needs to take noise into account. After a sharp decrease in the encoding error until  800 hidden units (see Figure S3), the performance starts saturating, especially for visual inputs. We therefore determined that 1500 hidden units would be a good trade-off between complexity and performance. Additionally, we performed the same analysis in the case of noiseless “up” passes, to see how quickly the network approaches the theoretical limit (that can only be achieved in the case of noiseless “up” passes) when it is not limited by noise.

Figure S3: Precision in the encoding of visual (left) and proprioceptive (right) inputs, as a function of the number of hidden units in the network. The encoding precision is defined as the standard deviation (average between x and y directions) of the positions obtained after generating unisensory inputs, encoding them in the multisensory layer and then decoding them again by projecting multisensory activity down to the unisensory layer and taking the barycentre of the generated activity. The ``down'' pass is always noiseless as it only acts as a decoding step, while we show results for both a noisy (solid blue line, used for determining the number of hidden units) and noiseless (dashed blue line) ``up'' pass. The maximal theoretical precision as obtained in Section 1 is shown as the black dashed line.

# Additional behavioural analyses

As mentioned in the main text, here we analyse the effect of the temporal delay of stimulation in more detail. First of all, we performed a Delay*Position*Congruency 3x2x3 ANOVA. Since there was no significant three-way interaction (p = .72), we pooled the two hand positions together, and performed a two-way Delay*Congruency ANOVA. We observed significant main effects of Congruency, as already confirmed by linear mixed models in the main text (F(2, 84) = 4.04, p = .0209). Moreover, we observed a significant main effect of Delay (F(2, 84) = 17.36, p < .001), possibly indicating overall expectation effects. Interestingly, the Delay*Congruency interaction was also significant ( F(4, 168) = 3.77, p = .0057 ), with an overall stronger effect of temporal delay (or distance) in the congruent condition (see Figure S4).

Figure S4: Multisensory facilitation plotted by congruency and by delay. In the congruent condition, the 'short' delay corresponds to approximately 10 cm from the hand, while the 'long' delay corresponds to 0 cm from the hand. Errorbars represent standard errors.
